# Polyamine and Its Metabolite H_2_O_2_ Play a Key Role in the Conversion of Embryogenic Callus into Somatic Embryos in Upland Cotton (*Gossypium hirsutum* L.)

**DOI:** 10.3389/fpls.2015.01063

**Published:** 2015-12-02

**Authors:** Wen-Han Cheng, Fan-Long Wang, Xin-Qi Cheng, Qian-Hao Zhu, Yu-Qiang Sun, Hua-Guo Zhu, Jie Sun

**Affiliations:** ^1^College of Agriculture/The Key Laboratory of Oasis Eco-Agriculture, Shihezi UniversityShihezi, China; ^2^Agriculture, Commonwealth Scientific and Industrial Research OrganisationCanberra, ACT, Australia; ^3^College of Life and Environmental Science, Hangzhou Normal UniversityHangzhou, China

**Keywords:** upland cotton, somatic embryogenesis (SE), polyamines (PAs), hydrogen peroxide (H_2_O_2_), polyamine oxidase (PAO), nitric oxide (NO)

## Abstract

The objective of this study was to increase understanding about the mechanism by which polyamines (PAs) promote the conversion of embryogenic calli (EC) into somatic embryos in cotton (*Gossypium hirsutum* L.). We measured the levels of endogenous PAs and H_2_O_2_, quantified the expression levels of genes involved in the PAs pathway at various stages of cotton somatic embryogenesis (SE), and investigated the effects of exogenous PAs and H_2_O_2_ on differentiation and development of EC. Putrescine (Put), spermidine (Spd), and spermine (Spm) significantly increased from the EC stage to the early phase of embryo differentiation. The levels of Put then decreased until the somatic embryo stage whereas Spd and Spm remained nearly the same. The expression profiles of *GhADC* genes were consistent with changes in Put during cotton SE. The H_2_O_2_ concentrations began to increase significantly at the EC stage, during which time both *GhPAO1* and *GhPAO4* expressions were highest and PAO activity was significantly increased. Exogenous Put, Spd, Spm, and H_2_O_2_ not only enhanced embryogenic callus growth and embryo formation, but also alleviated the effects of D-arginine and 1, 8-diamino-octane, which are inhibitors of PA synthesis and PAO activity. Overall, the results suggest that both PAs and their metabolic product H_2_O_2_ are essential for the conversion of EC into somatic embryos in cotton.

## Introduction

Plant regeneration through somatic embryogenesis (SE) has greatly aided gene transformation in many plant species, including cotton (*G. hirsutum* L.). The developmental stages involved in SE are analogous to those involved in zygotic embryogenesis (Silveira et al., 2013); however, somatic embryos have no endosperm and do not undergo some of the early divisions which are characteristic in zygotic embryos (Costa et al., 2014; Soriano et al., 2014). In cotton, plantlets have been regenerated via SE using various combinations of plant growth-regulators, such as 2, 4-dichlorophenoxyacetic acid (2, 4-D), indole-3-butyric acid (IBA), and naphthalene acetic acid (NAA) in combination with kinetin (KT) (Surgun et al., 2014). However, SE in cotton is often hampered by recalcitrance, long culture time, and high frequency of abnormal embryos. Low efficiency of regeneration is another major issue that limits the utilization of *Agrobacterium*-mediated transformation in cotton. Transcriptomic and proteomic studies have shown that genes and proteins involved in stress response, hormone metabolism, respiration, and photosynthesis play a role in increasing plantlet regeneration via SE in cotton (Ge et al., 2014). In addition, the effects of several genes on SE have been analyzed. For example, auxin-induced expression of *WUSCHEL* (*WUS*), a specifier of stem cell fate, was found to be essential for renewal of embryonic stem cells during SE in *Arabidopsis* (Su et al., 2009). *LEC2* plays critical roles during embryo development and is essential for induction of SE in *Arabidopsis* (Gaj et al., 2005). Understanding the molecular mechanisms of genes involved in SE and their interactions with other factors, such as hormones, can help with the initiation of embryogenic cultures and the enhancement of embryo yield and quality.

Polyamines (PAs), mainly putrescine (Put), spermidine (Spd), spermine (Spm), and the recently discovered thermospermine (T-Spm), are small, aliphatic amines that are widely present in all plant cells (Wojtasik et al., 2015). PAs are involved in many physiological processes, including cell division, rhizogenesis, senescence, floral development, fruit ripening, and the response to biotic and abiotic stress (Alcázar and Tiburcio, 2014). In plants, Put is synthesized by two pathways. One pathway begins with decarboxylation of arginine by arginine decarboxylase (ADC). PA concentrations are enhanced by exogenous arginine (Nieves et al., 2008). Another pathway begins with ornithine, which is converted into Put in a single-step reaction catalyzed by ornithine decarboxylase (ODC). Spermidine is synthesized by spermidine synthase (SPDS) through the addition of an aminopropyl moiety to Put. The aminopropyl moiety is donated by decarboxylated *S*-adenosylmethionine (dcSAM) which is converted from *S*-methylmethionine by *S*-adenosylmethionine decarboxylase (SAMDC). Spermidine then functions as a substrate to synthesize Spm by spermine synthase (SPMS). Thermospermine is an isomer of spermine and assumed to be synthesized by a mechanism analogous to that of Spd biosynthesis (Knott et al., 2007). L-arginine is an important substrate for the biosynthesis of PAs. However, L-arginine can also be used to generate nitric oxide (NO) by nitric oxide synthase (NOS) (Galea et al., 1996). Both H_2_O_2_ and NO are important signaling molecules involved in many developmental and physiological processes in plants. The H_2_O_2_ is involved in the regulation of root development, seed germination, programmed cell death, and defense responses to pathogen and abiotic stresses (Berna and Bernier, 1999). Studies indicate that H_2_O_2_ can be produced either by the NADPH-dependent pathway (Neill et al., 2002a), the antioxidant enzyme system (Alscher et al., 1997), or from PAs (mainly Put, Spd, and Spm) catalyzed by polyamine oxidase (PAO) or diamine oxidase (DAO). NO is a highly diffusible free radical that acts as an intra- and/or inter-cellular messenger to regulate various developmental and biological processes, including root development, seed germination, senescence, respiration, cell death, disease resistance, hormone responses, and abiotic stress responses.

Polyamines have previously been linked to both zygotic embryogenesis and SE. PA concentrations increase during the early stages of SE in conifers but decrease during the late stages (Gemperlová et al., 2009; Paul et al., 2009; Vuosku et al., 2012). Putrescine, spermidine, and spermine have also been shown to significantly improve SE of *Theobroma cacao* L, *Citrus sinensis* and *Hurst Ecotype* (Silva et al., 2009; Wu et al., 2009; Malá, 2012). Elevated levels of Put, Spd, and Spm in embryogenic cells, a result of enhanced expression of enzymes such as SAMDC, ADC and SPDS, suggest that PAs have a role in cellular differentiation during SE (Montague et al., 1978, 1979; Gemperlová et al., 2009; Niemenak et al., 2012). Both ADC mRNA and ADC protein were localized in dividing cells of embryo meristems, suggesting an association between ADC and mitosis (Vuosku et al., 2006). Exogenous Put and Spm enhanced the growth of embryogenic cultures of *Araucaria angustifolia* and significantly affected endogenous concentrations of PA, IAA and ABA in embryogenic tissues (Steiner et al., 2007). Nevertheless, an inverse correlation was observed between total free PA concentration and embryogenic potential in *Pinus nigra* Am. Sp. (Noceda et al., 2009). Biosynthesis of PAs is regulated by light in the presence of the plant growth regulators benzylaminopurine and cytokinin during SE in *C. canephora* (De-la-Peña et al., 2008). The dynamics of protein, sugar, starch, and amino acid were closely related to the accumulation of PAs during SE of *A. sellowiana* (Cangahuala-Inocente et al., 2014). In addition, genomic DNA of embryogenic tissues of *Pinus nigra* Am. Sp., i.e., those that are able to produce regenerated plantlets, was found to be lowly methylated (Noceda et al., 2009). These studies have investigated the effects of endogenous or exogenous PAs on SE in different species under different conditions; however, the physiological mechanisms by which PAs promote SE are still largely unclear.

*Gossypium hirsutum* L. cv. Xinluzao 33 is one of the main cotton cultivars used in Xinjiang Province, China. Regeneration of Xinluzao 33 *via* SE is problematic because of the lengthy time required for embryogenic callus induction and the low ratio of somatic embryo differentiation. The objectives of this study were (i) to enhance understanding about the relationship among PAs, H_2_O_2_, and NO during SE of Xinluzao 33, and (ii) to gain insight into the mechanisms by which PAs promote the conversion of embryogenic calli (EC) into somatic embryos.

## Materials and Methods

### Tissue Culture and Somatic Embryogenesis in Xinluzao 33

The cotton cultivar used in SE was Xinluzao 33, one of the major commercial cultivars in Xinjiang, China. The method for SE used in this study has been described previously (Sun et al., 2006). Briefly, Xinluzao 33 seeds were decoated, soaked in 0.1% (w/v) Hg_2_Cl_2_ for 10 min, and then rinsed three times with sterile, distilled water. The treated seeds were transferred to 100 mL Erlenmeyer flasks containing 25 mL of half-strength MS medium (Murashige and Skoog, 1962) and then incubated in the dark at 28°C for 7 days. Hypocotyls from 7-day-old sterile seedlings were cut into 1 cm segments and then transferred to callus-induction medium for SE. The culture mediums used for SE in this study are described in Supplementary Table S1. All cultures in this study were conducted under 16 h light: 8 h dark at 28°C.

### Establishment of Suspension Cultures for Uniform Embryogenic Callus

Embryonic callus (5 g) was collected and inoculated in liquid embryo induction medium, followed by shaking on an orbital shaker at 200 rpm in the dark at 28°C. After 5 days, the cultures were filtered into a flask through a 50-mesh sieve, and the supernatant was removed after 15-min sedimentation. The EC on the bottom of the flask were resuspended in 2 mL of liquid somatic embryo induction medium and used in various experiments.

### Exogenous PAs, H_2_O_2_, SNP, D-Arg, and 1, 8-DO Treatments

To determine the effects of PAs, H_2_O_2_, NO, PA synthesis inhibitor, and PAO inhibitor on the conversion of embryogenic callus into somatic embryos, uniform EC were cultured on somatic embryo induction medium supplemented with 1 mM of putrecine (Put), spermidine (Spd), spermine (Spm), sodium nitroprussiate (SNP; a NO donor) (Paulus et al., 1994), D-arginine (D-Arg, a specific PAs synthesis inhibitor) (Liu et al., 2006), H_2_O_2_, or 1,8-diamino-octane (1,8-DO, a specific PAO activity inhibitor) (Rodríguez et al., 2009). All reagents were purchased from Sigma Chemical Co (St. Louis, MO, USA). The 1 mM concentration was chosen based on preliminary gradient experiments (data not shown).

Uniform EC were inoculated at four positions (60 μL/position) in each Petri dish. Each treatment was replicated three times (i.e., three Petri dishes). The cultures were collected after 4 weeks, weighed, suspended in purified water, and then examined with a stereomicroscope to count embryos and cotyledonary embryos (Supplementary Figure S1). Increase in fresh weight (FW) during the culture period was determined by subtracting the initial callus weight from the total FW.

### Determination of the Free and Conjugated Polyamine Concentrations

Concentrations of free and conjugated PAs were determined using a modified high performance liquid chromatography (HPLC) method. Tissue samples (1 g) were collected at each stage of SE and then ground in liquid N_2_. The homogenate was resuspended in 5 mL 10% perchloric acid, incubated on ice for 1.5 h, and then centrifuged at 18514 *g* for 20 min at 4°C. Seven micro liter benzoyl chloride and 1 mL 2M NaOH were then added to 500 μL of the supernatant. The reactions were allowed to proceed at 37°C for 30 min and then 2 mL ether and 2 mL saturated NaCl were added to the reactions. The reactions were shaken for 5 min and then 1 mL of the ether phase was removed and dried under vacuum. The dried reactions were re-dissolved in 100 μL methanol before HPLC analysis. The HPLC was performed on an Agilent 1200 system (Agilent, USA) with an Agilent XDB-C18 (4.6 mm × 150 mm) column. The HPLC conditions were as follows: liquid phase with a methanol:water ratio of 60:40 (v/v), 1 mL/min of flow rate, 10 μL of sample per injection, detection at 30°C with a wave length of 254 nm, and 30 min of retention time. Peak areas and retention times were measured by comparison with standard Put, Spd, and Spm. The concentrations of PAs (ng of PAs g^-1^ fresh callus weight) were determined using a standard curve prepared with known amounts of standard Put, Spd, and Spm. The HPLC traces are shown in Supplementary Figure S2. The assays were technically repeated three times.

To determine the effects of Put, Spd, Spm and D-Arg on the PA concentrations, EC were cultured on liquid somatic embryo induction medium supplemented with 1 mM Put, Spd, Spm and D-Arg. The concentration of PAs in the EC was measured 3 days after inoculation. EC without any treatment were used as the control. The samples and treatments were replicated three times and the assays were technically repeated three times.

### Determination of H_2_O_2_ and NO

Hydrogen peroxide concentrations were determined with an H_2_O_2_ determination kit (Jiancheng Biochemistry Company, Nanjing, China) as previously described (Crumbliss et al., 1992). The absorbance of the titanium-peroxide complex was measured at 412 nm. The NO concentrations were determined using an NO determination kit (Jiancheng Biochemistry Company, Nanjing, China) as described by Qian et al. (2006). The absorbance was measured at 550 nm. The concentrations of H_2_O_2_ and NO (ng g^-1^ FW) in the samples were determined using a standard curve prepared with known amounts of H_2_O_2_ and NO. The assays were technically repeated three times.

To determine the effects of exogenous H_2_O_2_, Put, Spd, Spm, D-Arg and 1, 8-DO on H_2_O_2_ concentration, EC were cultured on liquid somatic embryo induction medium supplemented with 1 mM Put, Spd, Spm and D-Arg. The concentration of H_2_O_2_ in the EC was measured 3 days after inoculation. EC without any treatment were used as the control. The treatments were replicated three times and the assays were technically repeated three times.

### Detection of Hydrogen Peroxide by 3, 3′-Diaminobenzidine (DAB)

*In situ* detection of H_2_O_2_ was performed by DAB staining (Sigma–Aldrich) using a published method (Daudi et al., 2012). The staining reaction was terminated 5 h after DAB infiltration, and then the cultures were fixed in ethanol in a water bath at 95°C for 15 min. The cultures were reimmersed in bleach solution until the chlorophyll was completely bleached. The cultures were then visualized under white light and photographed. A combination of tools from ZEN Imaging Software (ZEISS, Germany) was used to establish the threshold of DAB staining in the cultures and to distinguish the staining from the background. The staining experiments were technically repeated three times.

### PAO Enzyme Activity Assay

Polyamine oxidase activity was determined using an ELISA Assay Kit (Jiancheng Biochemistry Company, Nanjing, China) according to the manufacturer’s instructions. The antibodies were produced by the Jiancheng Biochemistry Company (Nanjing, China). The PAs specificity of the PAO enzyme was evaluated by the OD value according to the known concentration of standard PAs. The tissue samples used for PAO enzyme assay were the same as those used for determining PA concentrations. The assays were technically repeated three times.

To determine the effects of Put, D-Arg, and 1,8-DO on PAO activity, EC were cultured on liquid somatic embryo induction medium supplemented with 1 mM Put, D-Arg or 1,8-DO. The PAO activities in the EC were measured 3 days after inoculation. EC without any treatment were used as the control. The treatments were replicated three times and the assays were technically repeated three times.

### Gene Identification

To quantify the expression levels of cotton genes encoding ADC, PAO and SAMDC at various stages of cotton SE, protein sequences of the *Arabidopsis* orthologs [AtADC (NM_127204), AtPAO (NM_121373) and AtSAMDC1 (NC_003074.8)] were used as query to search against the *G. raimondii* genome (Paterson et al., 2012) using an *E*-value of 0.99. This analysis identified three ADC-encoding genes, four PAO-encoding genes, and four SAMDC-encoding genes. These genes were used to investigate the expression levels of their orthologs in *G. hirsutum*. The genes encoding SPDS, SPMS, CAT, NOX, SOD, APX and NOS were determined based on our transcriptome sequencing of upland cotton *cv.* Xinluzao 33 during SE. The nucleotide sequences of these genes and their gene ID are listed in Supplementary Table S2.

### RNA Extraction and Quantitative Real Time PCR

Total RNA was extracted from the samples using a modified CTAB method (Chang et al., 1993). Approximately 2 μg of total RNA was reverse transcribed into cDNA using the Prime Script RT reagent kit (Takara, Japan) with gDNA Eraser (Takara, Japan). The cDNA templates were diluted ten times prior to qPCR. The qRT-PCR experiment was conducted in a Roche LightCycler 480 system (Roche, Switzerland) using the SYBR Premix ExTaqTM kit (Takara, Japan) and the following thermal cycling program: pre-incubation at 95°C for 2 min, followed by 40 cycles of 94°C for 15 s, 56°C for 20 s, and 72°C for 20 s. The relative expression levels were determined using 2^-ΔCt^ with the ubiquitin (*GhUBI*, XM_012634824) gene as the reference. The primers used in qRT-PCR were designed using Primer Premier 5.0 (Supplementary Table S3). The expression assay of each sample was performed using three biological replicates and each biological replicate was technically repeated three times.

### Statistical Analysis

Analysis of variance was performed using SPSS16.0 statistical analysis package. Differences between means were compared by Fisher’s least-significant-difference test at the 5 and 1% probability level.

## Results

### Polyamines Significantly Increased between the Embryogenic Callus and Early Embryo Differentiation Stages

**FIGURE 1:**
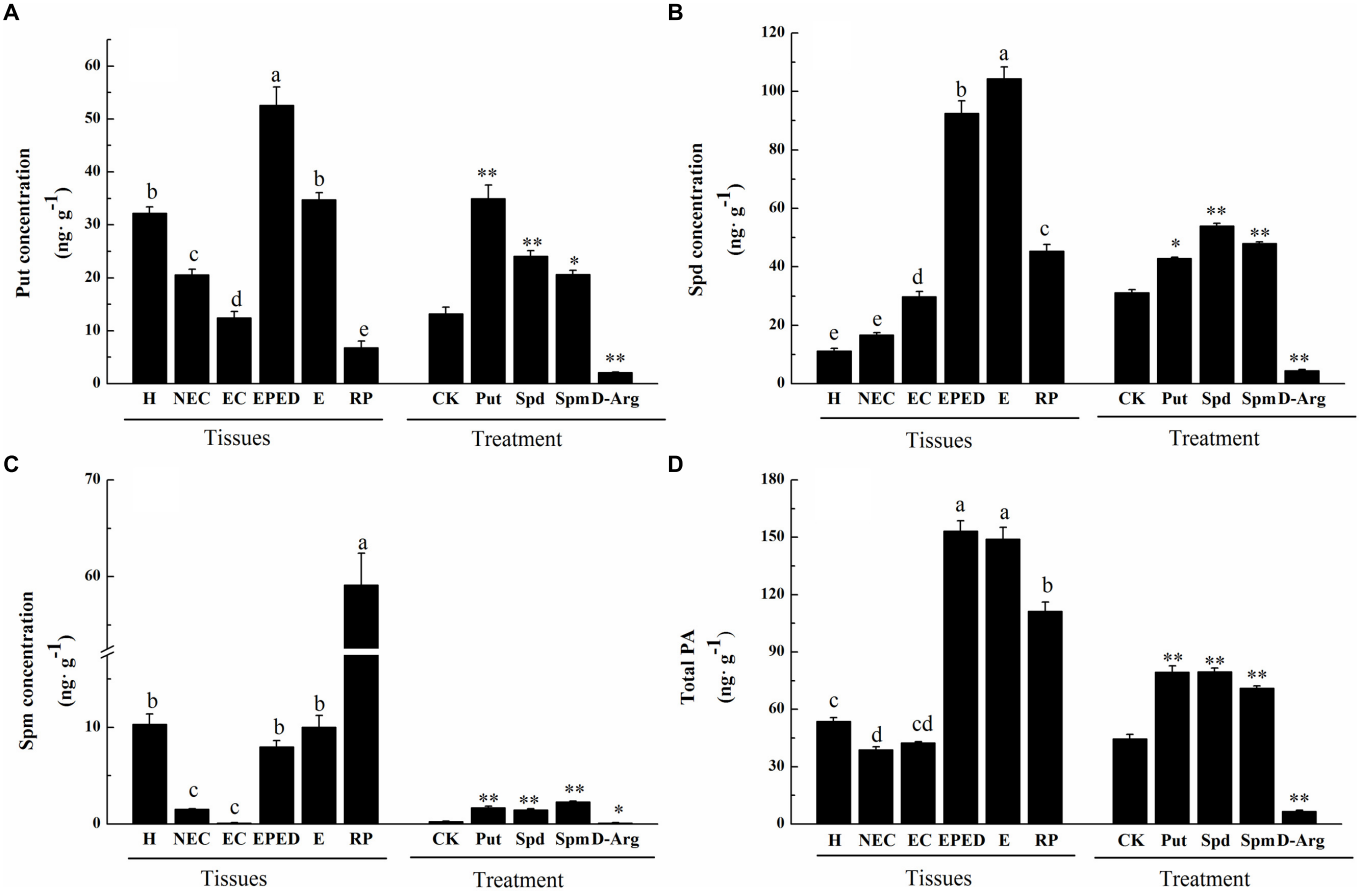
**Polyamine concentrations at different stages of somatic embryogenesis (SE) in ‘Xinluzao 33’ and the effects of different chemical treatments on polyamine concentrations. (A)** Putrescine concentration. **(B)** Spermidine concentration. **(C)** Spermine concentration. **(D)** Total PA. Abbreviations on the *x*-axis: H, hypocotyl; NEC, non-embryogenic callus; EC, embryogenic callus; EPED, early phase of embryo differentiation; E, embryo; RP, regenerated plantlets. Treatment on the *x*-axis, CK, control; D-Arg, D-arginine treatment; Put, putrescine treatment; Spd, spermidine treatment; Spm, spermine treatment. Values are the mean + standard error (*n* = 3). Different lowercase letters above the bars indicate significant differences at *P* < 0.05 according to LSD multiple range test. ^∗^ and ^∗∗^ indicate significant differences compared with the control at *P* < 0.05 and *P* < 0.01, respectively, according to LSD multiple range test.

Generally, cotton SE includes four stages, i.e., callus induction, embryogenic callus induction, embryo differentiation, and plant regeneration. To investigate temporal changes of free and conjugated PAs during cotton SE, the concentrations of three common PAs (Put, Spd, and Spm) were analyzed in hypocotyl-derived explants and in samples collected at different stages of cotton SE (Supplementary Figure S3). Compared to non-embryogenic callus, embryogenic callus showed a significant decrease in Put, a significant increase in Spd, and no changed in Spm (**Figures 1A–C**). Compared to embryogenic callus, a significant increase in all three PAs (Put: 4.2-fold; Spd: 3.1-fold; Spm: 8.1-fold) was observed in the early phase of embryo differentiation (**Figures 1A–C**). The total concentration of PAs increased 3.6-fold from the embryogenic callus stage to the early phase of embryo differentiation (**Figure 1D**). The concentrations of these PAs remained high in somatic embryos. The concentrations of Put and Spd declined significantly in the regenerated plantlets whereas the concentration of Spm increased significantly in the regenerated plantlets (**Figures 1A–C**). Compared to Put and Spd, Spm concentrations remained relatively low throughout most of the SE stages except in the regenerated plantlets. As expected, supplementation of exogenous Put, Spm or Spd significantly increased endogenous Put, Spm and Spd in embryogenic callus. Application of D-Arg, an inhibitor of PA synthesis, significantly decreased endogenous Put, Spm and Spd in embryogenic callus (**Figure 1**).

**FIGURE 2:**
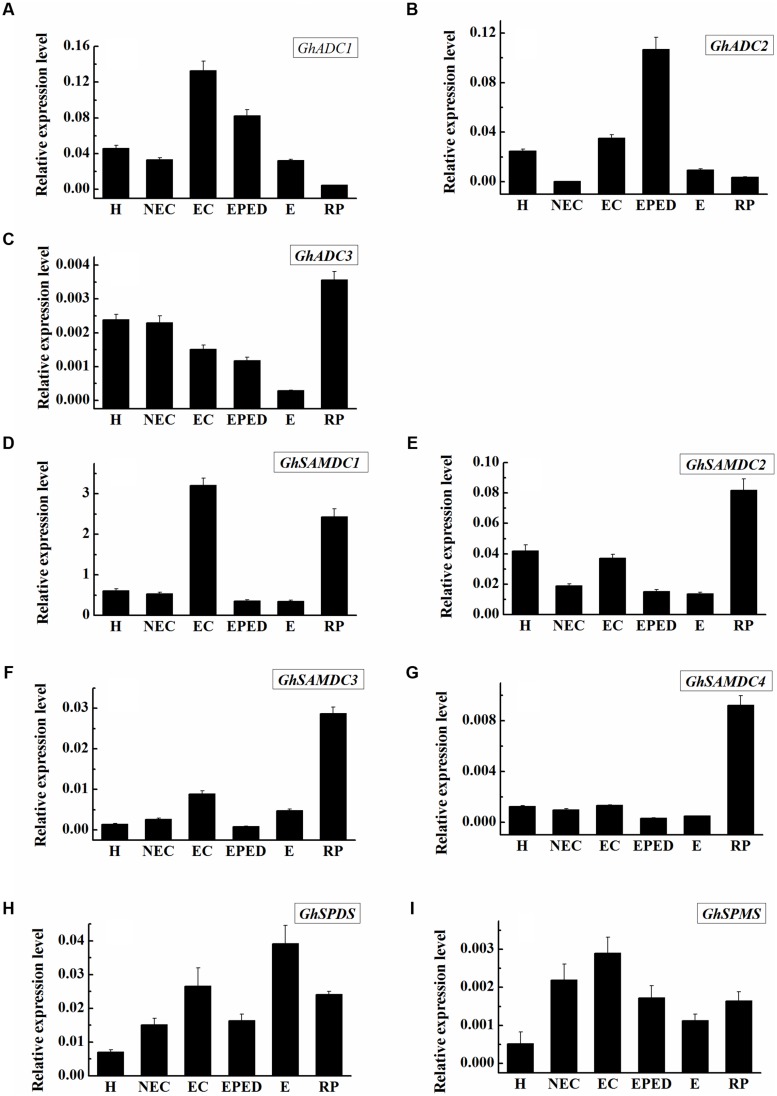
**Expression level of genes encoding *SAMDC*, *ADC*, *SPDS* and *SPMS* during SE in ‘Xinluzao 33’.** The equation 2^-ΔCt^ was applied to calculate the relative expression level using GhUBI as the reference gene. **(A–I)** Relative expression level of GhSAMDC1/2/3/4, GhADC1/2/3, GhSPDS and GhSPMS. **(A)** Relative expression level of GhADC1. **(B)** Relative expression level of GhADC2. **(C)** Relative expression level of GhADC3. **(D)** Relative expression level of GhSAMDC1. **(E)** Relative expression level of GhSAMDC2. **(F)** Relative expression level of GhSAMDC3. **(G)** Relative expression level of GhSAMDC4. **(H)** Relative expression level of GhSPDS. **(I)** Relative expression level of GhSPMS. Abbreviations on the *x*-axis: H, Hypocotyl; NEC, Non-embryogenic callus; EC, Embryogenic callus; EPED, Early phase of embryo differentiation; E, Embryo; RP, Regenerated plantlets. Values are the mean + standard error (*n* = 3).

Arginine decarboxylase (ADC) catalyzes the first reaction toward biosynthesis of Put. *S*-adenosylmethionine decarboxylase (SAMDC) converts dcSAM into Spd by adding an aminopropyl moiety to Put. The main genes for synthesis of Spd and Spm from Put are *SPDS* and *SPMS*. We analyzed the expression levels of the genes encoding ADC, SAMDC, SPDS and SPMS at different stages during cotton SE. Of the three ADC-encoding genes, the expression level of *GhADC3* was much lower than that of *GhADC1* and *GhADC2*, suggesting that *GhADC1* and *GhADC2* are the major genes responsible for the biosynthesis of Put in cotton callus tissue. The expression levels of *GhADC1* and *GhADC2* were highest at the embryogenic callus stage and the early phase of embryo differentiation. There was no clear correlation between the individual expression levels and Put (**Figures 2A–C**). This suggested that Put concentrations might be determined by the combined action of all *GhADC* genes. Among the four *GhSAMDC* genes, the expression of *GhSAMDC1* was higher than that of *GhSAMDC2*, *GhSAMDC3* and *GhSAMDC4* in all the samples. The highest *GhSAMDC1* expression level was in the embryogenic callus, whereas the highest expression of the other three *GhSAMDC* genes was in regenerated plantlets (**Figures 2D–G**). The highest *SPDS* and *SPMS* expression was in the somatic embryos and embryogenic callus, respectively (**Figures 2H,I**). Although Spd increased significantly at the early phase of embryo differentiation and the somatic embryo stage, the expression levels of all four *GhSAMDC* genes were relatively low at these two stages (**Figure 1B**). These results suggest that Spd concentrations may be determined by Spd synthase (SPDS) rather than by SAMDC.

### Exogenous Polyamines Promoted the Conversion of Embryogenic Callus into Somatic Embryos

**FIGURE 3:**
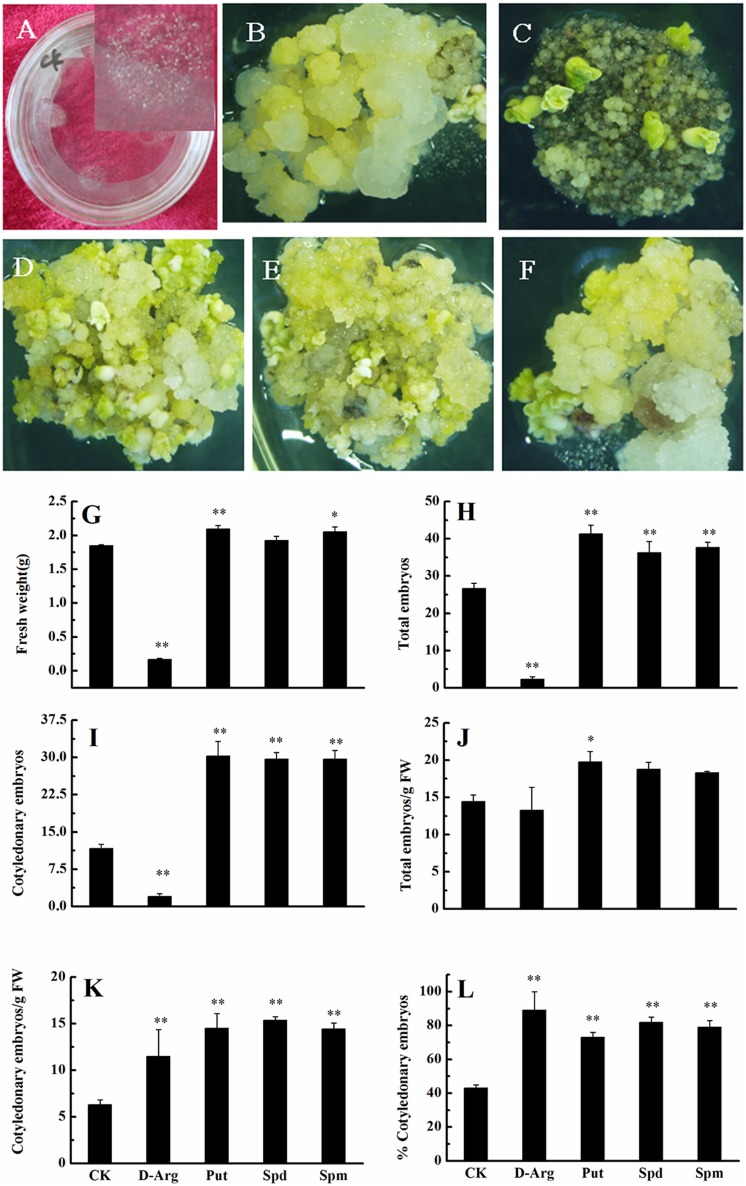
**Effects of exogenous PA and D-arg on the conversion of embryogenic callus into somatic embryos in ‘Xinluzao 33.’ (A)** Initial status of embryogenic callus after passing through a 50 mesh sieve. **(B–F)** Status of embryogenic callus after 30 days on the somatic embryo induction medium. **(B)** Untreated medium (control). **(C)** D-arginine treatment. **(D)** Put treatment. **(E)** Spd treatment. **(F)** Spm treatment. **(G–L)** Statistic analysis of different treatments. **(G)** Fresh weight (g). **(H)** Total embryo number. **(I)** Cotyledonary embryo number. **(J)** Total embryo embryo number/g FW. **(K)** Cotyledonary embryo number/g FW. **(L)** % Cotyledonary embryos. Abbreviations on the *x*-axes: CK, control; D-Arg, D-arginine treatment; Put, putrescine treatment; Spd, spermidine treatment; Spm, spermine treatment. Data are mean + standard error (*n* = 3). ^∗^ and ^∗∗^ indicate significant differences compared with the control at *P* < 0.05 and *P* < 0.01, respectively, according to LSD multiple range test.

The above results showed that free and conjugated PA concentrations increased significantly during the early phase of embryo differentiation; therefore, our next experiments focused on this stage. The effect of PAs on the conversion of embryogenic callus into somatic embryos was studied by culturing EC on somatic embryo induction medium supplemented with D-Arg (an inhibitor of PAs synthesis), Put, Spd, or Spm. After 4 weeks of cultivation, calli looked moist in the control (i.e., no treatment) (**Figures 3A,B**). The D-Arg significantly inhibited the embryonic callus growth (**Figure 3C**), whereas exogenous PAs promoted callus growth (**Figures 3D–F**). Compared with the control, D-Arg significantly reduced FW, total embryo number, and the number of cotyledonary embryos (**Figures 3G–I**). In contrast, D-Arg increased the number of cotyledonary embryos/g FW and the percentage of cotyledonary embryos (**Figures 3K,L**). One explanation is that D-Arg treatment increased the survival rates of embryos. Compared with the control, the three PA treatments significantly increased (∼1.5 fold, *P* < 0.05) the total embryo number, the number of cotyledonary embryos, the cotylendonary embryo number/g FW, and the percentage of cotyledonary embryos (**Figures 3H,I,K,L**). The Put treatment also significantly increased the tissue FW and the total embryo number/g FW compared with the control (**Figures 3G,J**). Overall, these data indicated that application of exogenous PAs promoted the conversion of embryogenic callus into somatic embryos.

### Effects of H_2_O_2_ and NO on the Conversion of Embryogenic Callus into Somatic Embryos in Cotton

**FIGURE 4:**
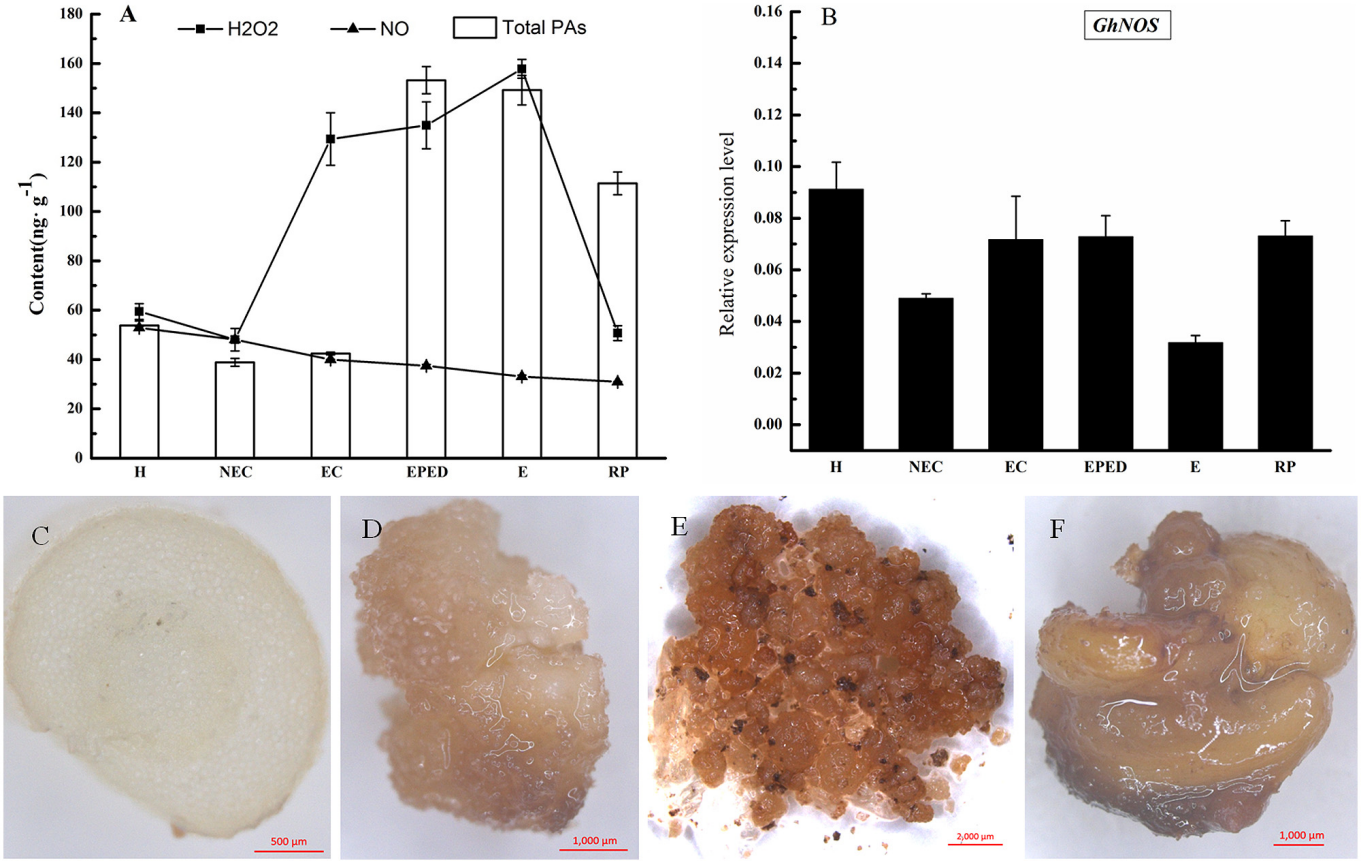
**Determination of H_2_O_2_ and NO levels during SE in ‘Xinluzao 33.’ (A)** The concentration of H_2_O_2_, NO, and total PA at different stages of SE. Abbreviations on the *x*-axis: H, hypocotyl; NEC, non-embryogenic callus; EC, embryogenic callus; EPED, early phase of embryo differentiation; E, embryo; RP, regenerated plantlets. **(B)** Expression level of *GhNOS* during SE. **(C–F)** Detection of hydrogen peroxide by DAB. **(C)** Transverse sliced hypocotyl. **(D)** Non-embryogenic callus; **(E)** Embryogenic callus; **(F)** Somatic embryos.

The next step in our study was to determine the role of two signaling molecules in the PAs metabolic pathway (H_2_O_2_ and NO) on the conversion of EC into somatic embryos. The concentration of endogenous H_2_O_2_ increased 2.7-fold from the non-embryogenic callus stage to the embryogenic callus stage. Endogenous H_2_O_2_ concentrations remained high during the early phase of embryo differentiation and the embryo stage and then significantly declined in the regenerated plantlets. Endogenous H_2_O_2_ concentrations were greatest at the somatic embryo stage, a critical stage of cotton SE. The temporal changes in H_2_O_2_ concentrations were similar to the changes in the concentrations PAs. There was significant positive correlation (*r* = 0.56, *P* < 0.05) between the concentrations of PAs and H_2_O_2_ (**Figure 4A**).

Diaminobenzidine (DAB) can be oxidized by H_2_O_2_ to give a dark-brown color. We used DAB staining to visualize the presence and activity of H_2_O_2_ in hypocotyl, non-EC, EC and somatic embryos. EC and somatic embryos were stained deep brown (**Figures 4E,F**), whereas staining in hypocotyls and non-EC were much lighter (**Figures 4C,D**). These results confirmed a relatively high level of endogenous H_2_O_2_ in the EC and somatic embryos. The DAB staining in hypocotyls and non-EC did not strictly match the H_2_O_2_ concentrations, probably due to biased sampling of un-uniform non-EC. In contrast, endogenous NO concentrations did not change significantly during cotton SE (**Figure 4A**). This was consistent with the relatively stable expression level of *GhNOS* during SE (**Figure 4B**). There was no significant correlation between the concentrations of PAs and NO.

**FIGURE 5:**
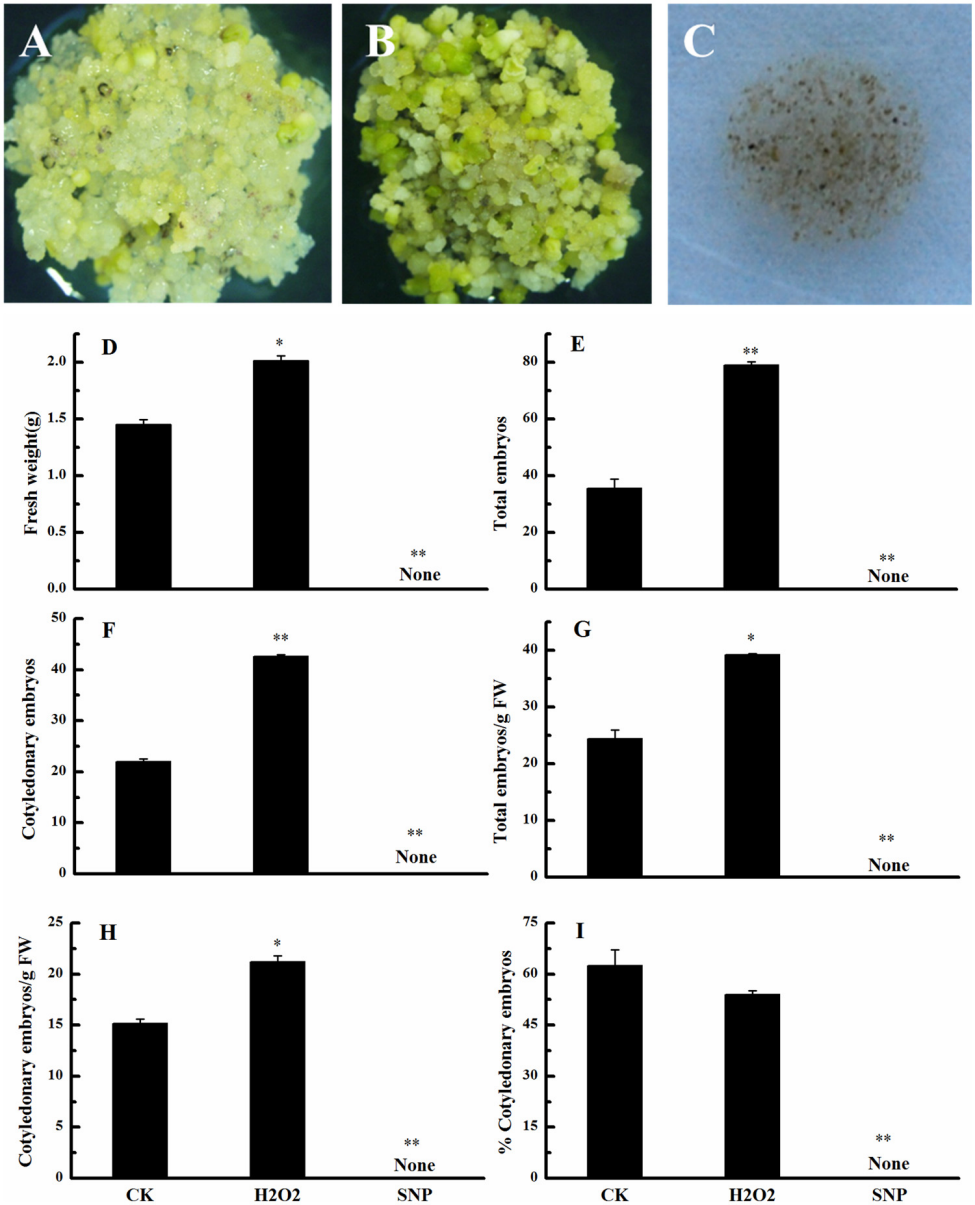
**Effects of exogenous H_2_O_2_ and NO on the conversion of embryogenic callus into somatic embryos in ‘Xinluzao 33.’ (A–C)** Status of embryogenic callus after 30 days on somatic embryo induction medium. **(A)** Untreated medium (CK). **(B)** H_2_O_2_ treatment. **(C)** Sodium nitroprussiate (SNP, a NO donor) treatment. **(D–I)** Statistical analysis of the treatments. **(D)** Fresh weight (g). **(E)** Total embryo number. **(F)** Cotyledonary embryo number. **(G)** Total embryo number/g FW. **(H)** Cotyledonary embryo number/g FW. **(I)** % Cotyledonary embryos. Abbreviations on the *x*-axes: CK, control; H2O2, H_2_O_2_ treatment; SNP, sodium nitroprussiate treatment. Data are mean + standard error (*n* = 3). ^∗^ and ^∗∗^ indicate significant differences at *P* < 0.05 and *P* < 0.01, respectively, according to LSD multiple range test.

We also investigated the effects of exogenous H_2_O_2_ and SNP (an NO donor) on the conversion of embryogenic callus into somatic embryos. Compared with the control, exogenous H_2_O_2_ significantly promoted development of the culture (**Figures 5A,B**), whereas SNP inhibited development (**Figure 5C**). Exogenous H_2_O_2_ significantly increased the FW, the total number of embryos, the number of cotyledonary embryos, total embryo number/g FW, and cotyledonary embryo number/g FW (**Figures 5D–H**), but had no significant effect on the percentage of cotyledonary embryos (**Figure 5I**). These results suggested that similar to its metabolic precursor (i.e., PAs), H_2_O_2_ promotes the conversion of embryogenic callus into somatic embryos in cotton. Few EC were observed in the SNP treatment. This observation, along with the observation that NO concentrations did not change during cotton SE, suggests that NO has a negative role in cotton SE.

### H_2_O_2_ Alleviated the Inhibitory Effect of D-Arg and Promoted the Conversion of Embryogenic Callus into Somatic Embryos

**FIGURE 6:**
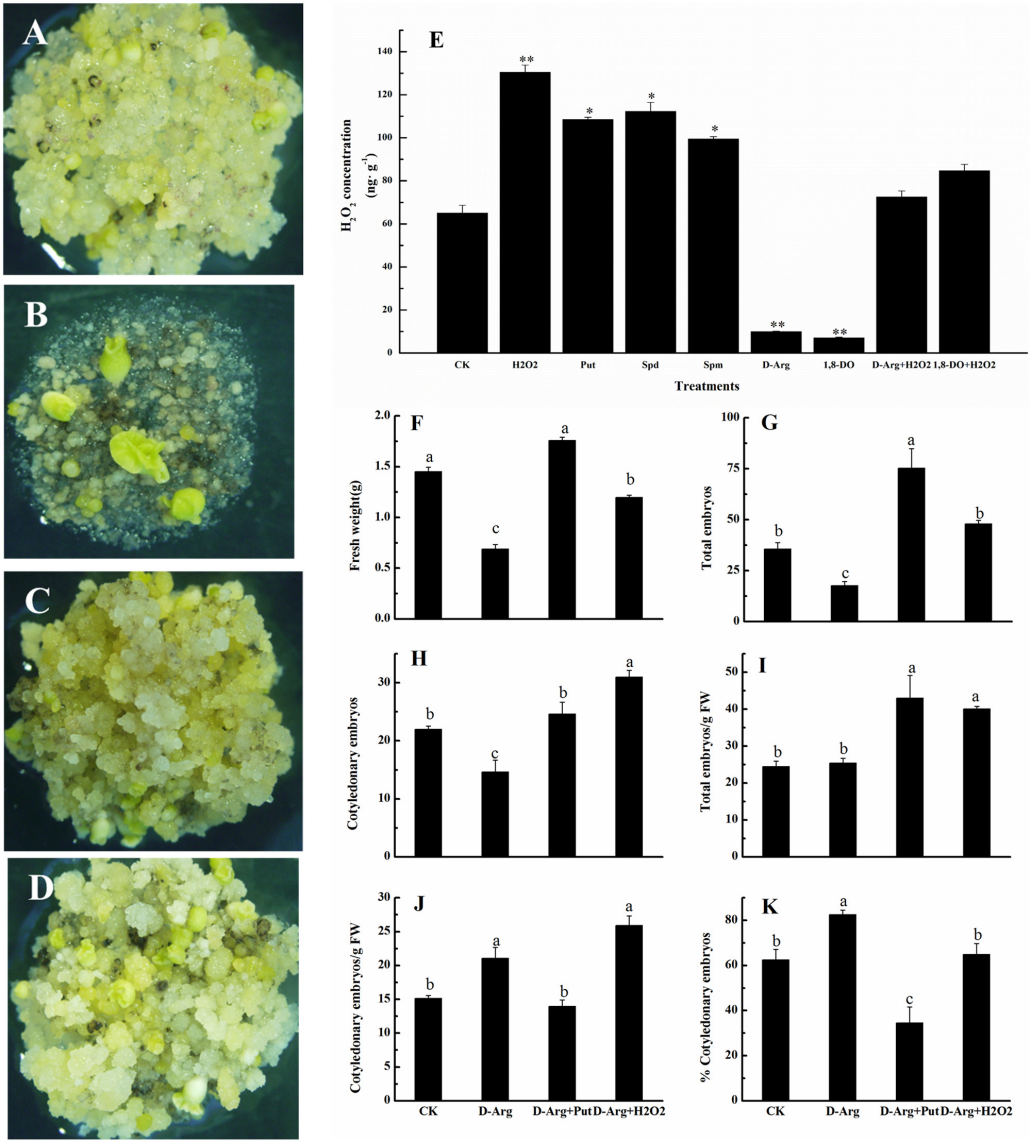
**H_2_O_2_ alleviated the inhibitory effect of D-Arg and promoted the conversion of embryogenic callus into somatic embryos in ‘Xinluzao 33.’ (A–D)** Status of embryogenic callus after 30 days on the media. **(A)** Status of embryogenic callus after 30 days on the untreated media as control. **(B)** D-arginine treatment. **(C)** D-arginine + Put treatment. **(D)** D-arginine + H_2_O_2_ treatment. **(E)** H_2_O_2_ concentration in the H_2_O_2_, Put, Spd, Spm, D-Arg, 1, 8-DO, D-Arg + H_2_O_2_ and 1, 8-DO + H_2_O_2_ treatments. ^∗^ and ^∗∗^ indicate significant differences at *P* < 0.05 and *P* < 0.01, respectively, according to LSD multiple range test. **(F–K)** Statistical analysis of the treatments. **(F)** Fresh weight (g). **(G)** Total embryos. **(H)** Cotyledonary embryos. **(I)** Total embryos/g FW. **(J)** Cotyledonary embryos/g FW. **(K)** % Cotyledonary embryos. The horizontal axis of **(F–K)** were same, CK: control, D-Arg: D-arginine treatment, D-Arg + Put: media supplement with D-arginine and putrescine, D-Arg + H_2_O_2_: media supplemented with D-arginine and H_2_O_2_. Values are the mean + standard error (*n* = 3). Different lowercase letters above the bars indicate significant differences at *P* < 0.05 according to LSD multiple range tests.

To learn more about the effects of H_2_O_2_ and PAs on the conversion of embryogenic callus into somatic embryos, we cultured EC on somatic embryo induction medium containing D-Arg which is an inhibitor of PAs synthesis. Compared with the control (**Figure 6A**), D-Arg significantly suppressed embryonic callus growth (**Figure 6B**). The callus FW, the total embryo number, and the number of cotyledonary embryos were significantly less in the D-Arg treatment than in the control (**Figures 6F–H**). However, the suppressive effects of D-Arg were alleviated when either Put or H_2_O_2_ was added to the medium (**Figures 6C,D**). The callus FW, the total embryo number, the number of cotyledonary embryos, and total embryo number/g FW were significantly higher in the D-Arg + Put and D-Arg + H_2_O_2_ treatments than in the D-Arg treatment (**Figures 6F–I**). Compared with the control, D-Arg increased the number of cotyledonary embryos/g FW and the percentage of cotyledonary embryos. This was similar to the results shown in **Figures 3K,L**. However, D-Arg + Put and D-Arg + H_2_O_2_ had little effect on these variables (**Figures 6J,K**). It should be noted that the effects of Put and H_2_O_2_ on the formation of somatic embryos were not exactly the same. The Put seemed to promote embryos formation whereas H_2_O_2_ seemed to promote cotyledonary embryos development (**Figures 6G,H**). These results further confirmed a role of H_2_O_2_ in the conversion of embryogenic callus into somatic embryos.

Concentration of H_2_O_2_ was also measured in EC growing on medium supplemented with H_2_O_2_, Put, Spd, Spm, D-Arg, 1, 8-DO, D-Arg + H_2_O_2_ and 1, 8-DO + H_2_O_2_. The results showed that H_2_O_2_, Put, Spd, or Spm significantly increased H_2_O_2_ concentrations, whereas D-Arg and 1, 8-DO significantly reduced H_2_O_2_ concentrations. Supplementation of H_2_O_2_ in the D-Arg or 1, 8-DO-containing medium alleviated the inhibitory effects of D-Arg and 1, 8-DO on H_2_O_2_ production in EC (**Figure 6E**). These data indicated that accumulation of PAs leads to greater H_2_O_2_ concentrations. Furthermore, H_2_O_2_ concentrations are reduced by inhibitors of the synthesis or oxidative metabolism. The levels of PA and H_2_O_2_ are closely related.

### Polyamine Oxidase (PAO) Plays a Crucial and Positive Role During the Conversion of Embryogenic Callus into Somatic Embryos

**FIGURE 7:**
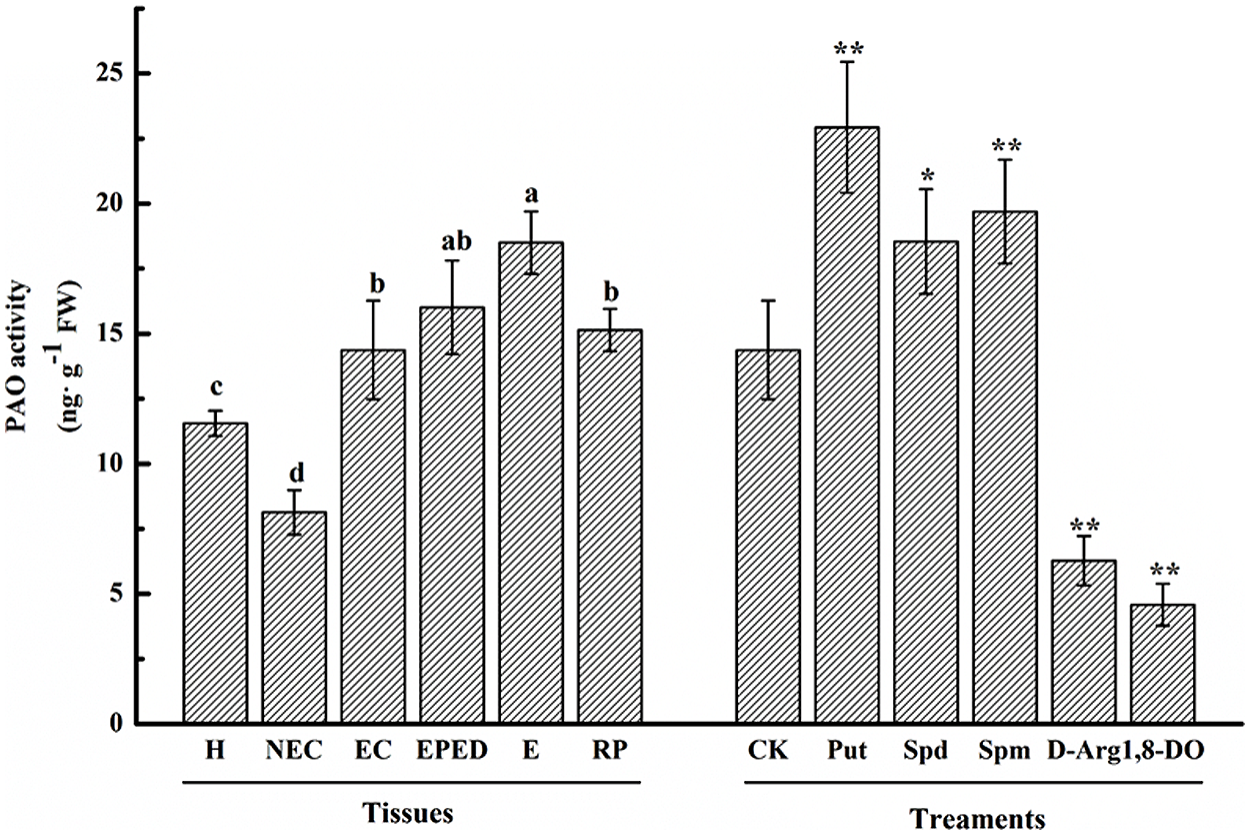
**Polyamine oxidase activity during SE of ‘Xinluzao 33.’** The horizontal axis tissues H, hypocotyl; NEC, non-embryogenic callus; EC, embryogenic callus; EPED, early phase of embryo differentiation; E, embryo; RP, regenerated plantlets. The treatments are liquid embryo induction media supplemented with Put, Spd, Spm, D-arg and 1, 8-DO 3 days after inoculation of embryogenic callus. CK is unamended induction medium. Values are the mean + standard error (*n* = 3). Different lowercase letters above the bars indicated significant differences in PAO activity among the stages at *P* < 0.05. ^∗^ and ^∗∗^ indicate significant differences at *P* < 0.05 and *P* < 0.01, respectively, according to LSD multiple range test.

Polyamine oxidase catalyzes the oxidation of PAs to produce H_2_O_2_. To investigate the relationship between the activity of PAO and SE, we analyzed the PAO enzyme activities in the various SE stages by ELISA assay. Compared with hypocotyls, PAO activity was significantly reduced in non-embryogenic callus, whereas PAO activity was significantly increased in embryogenic callus, the early phase of differentiated embryos, the somatic embryos and the regenerated plantlets (**Figure 7**). The highest PAO activity was observed at the somatic embryo stage, which is one of the most important and problematic stages in cotton SE (**Figure 7**). The PAO activity increased by 1.2, 1.3, and 1.6-fold in Spd-, Spm-, and Put-treated EC, respectively, whereas PAO activity was significantly reduced in the D-Arg- and 1, 8-DO-treated EC (**Figure 7**).

**FIGURE 8:**
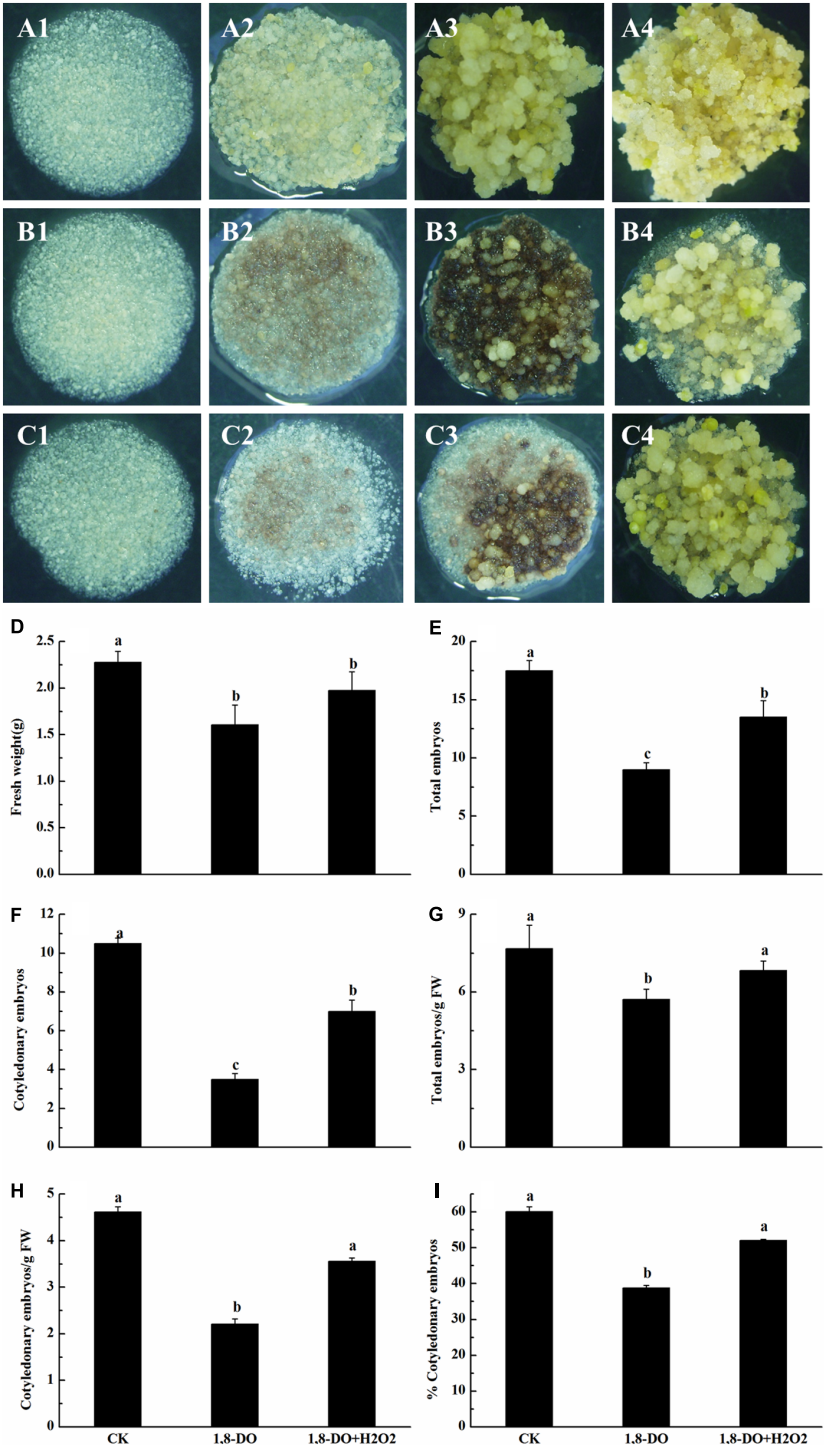
**Polyamine oxidase influenced the conversion of embryogenic callus into somatic embryos of ‘Xinluzao 33.’ (A1–4 to C1–4)** Represent the status of embryogenic callus on the media at different times during the culture (1: 0 days; 2: 3 days; 3: 10 days; 4: 30 days). **(A)** Status of embryogenic callus on the untreated media (control). **(B)** 1, 8-DO treatment. **(C)** 1, 8-DO + H_2_O_2_ treatment. The cultures in 1, 8-DO exhibited browning and underwent necrosis early in the incubation, probably due to loss of efficiency of 1, 8-DO. The cultures recovered after about 10 days and then grew better. **(D–I)** Statistical analysis of different treatments. **(D)** Fresh weight (g). **(E)** Total embryos. **(F)** Cotyledonary embryos. **(G)** Total embryos/g FW. **(H)** Cotyledonary embryos/g FW. **(I)** % Cotyledonary embryos. The horizontal axis of D-I were same, CK: control, 1, 8-DO: 1, 8-diamino-octane treatment, 1, 8-DO + H_2_O_2_: media supplement with 1, 8-diamino-octane and H_2_O_2_. Values are the mean + standard error (*n* = 3). Different lowercase letters above the bars indicate significant differences at *P* < 0.05 according to LSD multiple range test.

When grown on somatic embryo 0induction medium containing 1, 8-DO, the cultures showed browning and necrosis (**Figures 8A,B**). There was also a significant decrease in callus FW, total embryo number, cotyledonary embryo number, total embryo number/g FW, cotyledonary embryo number/g FW, and percentage of cotyledonary embryos. These negative effects of 1, 8-DO were lessened when H_2_O_2_ was added to the 1, 8-DO-containing medium, with the exception of FW (**Figures 8C–I**). This was largely due to increased H_2_O_2_ concentrations (84.6 g⋅ g^-1^ FW) in EC grown on 1, 8-DO+H_2_O_2_ medium (**Figure 6E**). These results suggested that the conversion of EC into somatic embryos was significantly reduced when PAO activity was inhibited. The effect was alleviated by application of exogenous H_2_O_2_. These results also suggested that PAO-catalyzed production of H_2_O_2_ plays a crucial and positive role in the conversion of embryogenic callus into somatic embryos in cotton.

**FIGURE 9:**
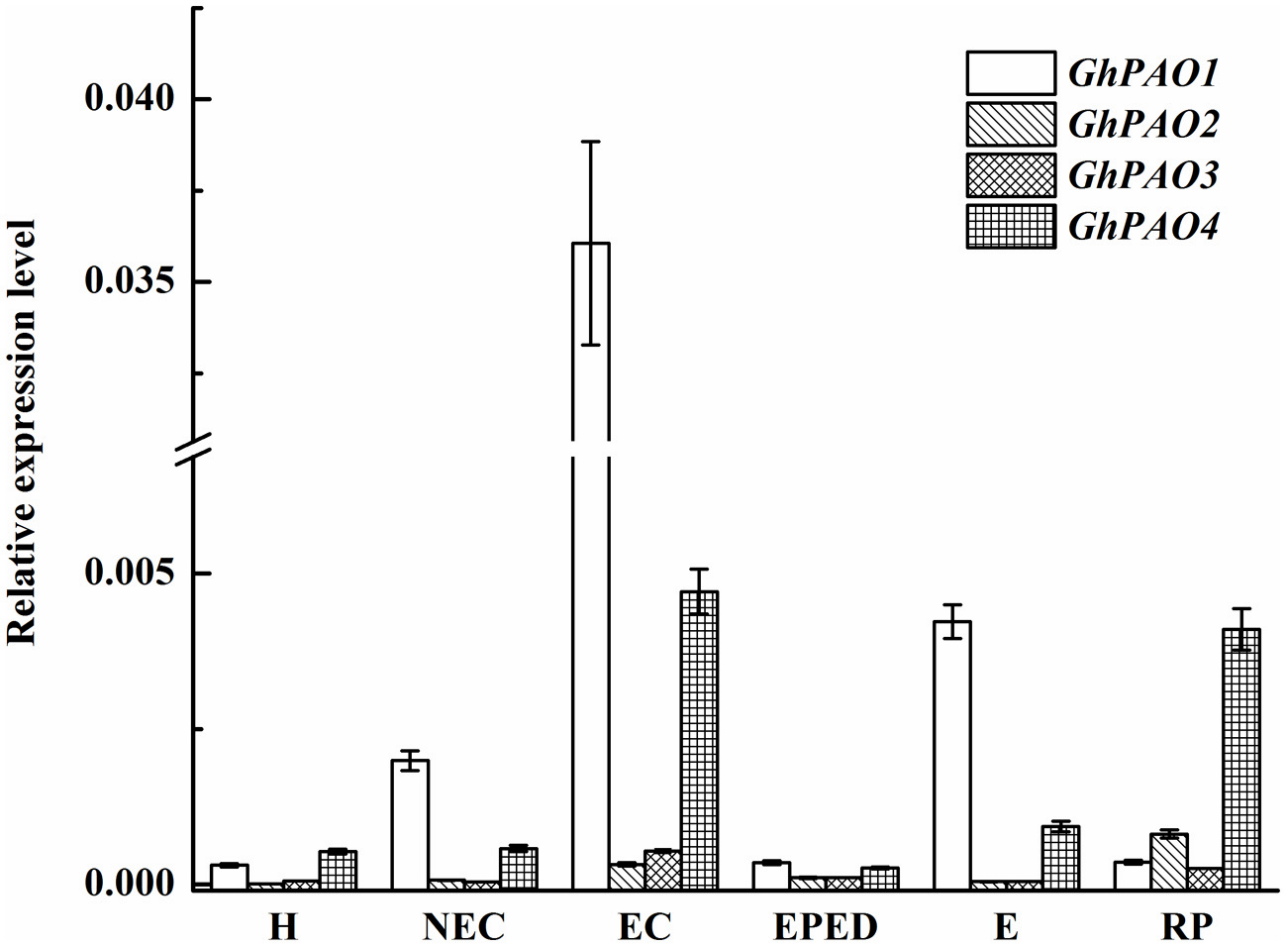
**Relative expression of *GhPAO1-4* during SE in ‘Xinluzao 33.’** The equation 2^-ΔCt^ was applied to calculate the relative expression level using *GhUBI* as the reference gene. The horizontal axis: H, hypocotyl; NEC, non-embryogenic callus; EC, embryogenic callus; EPED, early phase of embryo differentiation; E, embryo; RP, regenerated plantlets. Values are the mean + standard error (*n* = 3).

The *G. hirsutum* genome contains four PAO-encoding genes. *GhPAO1* and *GhPAO4* were expressed at higher levels than *GhPAO2* and *GhPAO3* in hypocotyls as well as all callus and embryonic samples (**Figure 9**). The expression levels of *GhPAO1* were consistently higher than that of *GhPAO4* in non-embryogenic callus, embryogenic callus, embryos at early phase of differentiation, and somatic embryos, the expression levels of both *GhPAO1* and *GhPAO4* increased dramatically from the non-embryogenic callus stage to the embryogenic callus stage. *GhPAO1* and *GhPAO4* expression was highest in embryogenic callus, although a relatively high level of *GhPAO1* and *GhPAO4* was also observed in somatic embryos and regenerated plantlets (**Figure 9**). These results suggested that both *GhPAO1* and *GhPAO4* may play a crucial role in the generation and differentiation of embryogenic callus during cotton SE.

**FIGURE 10:**
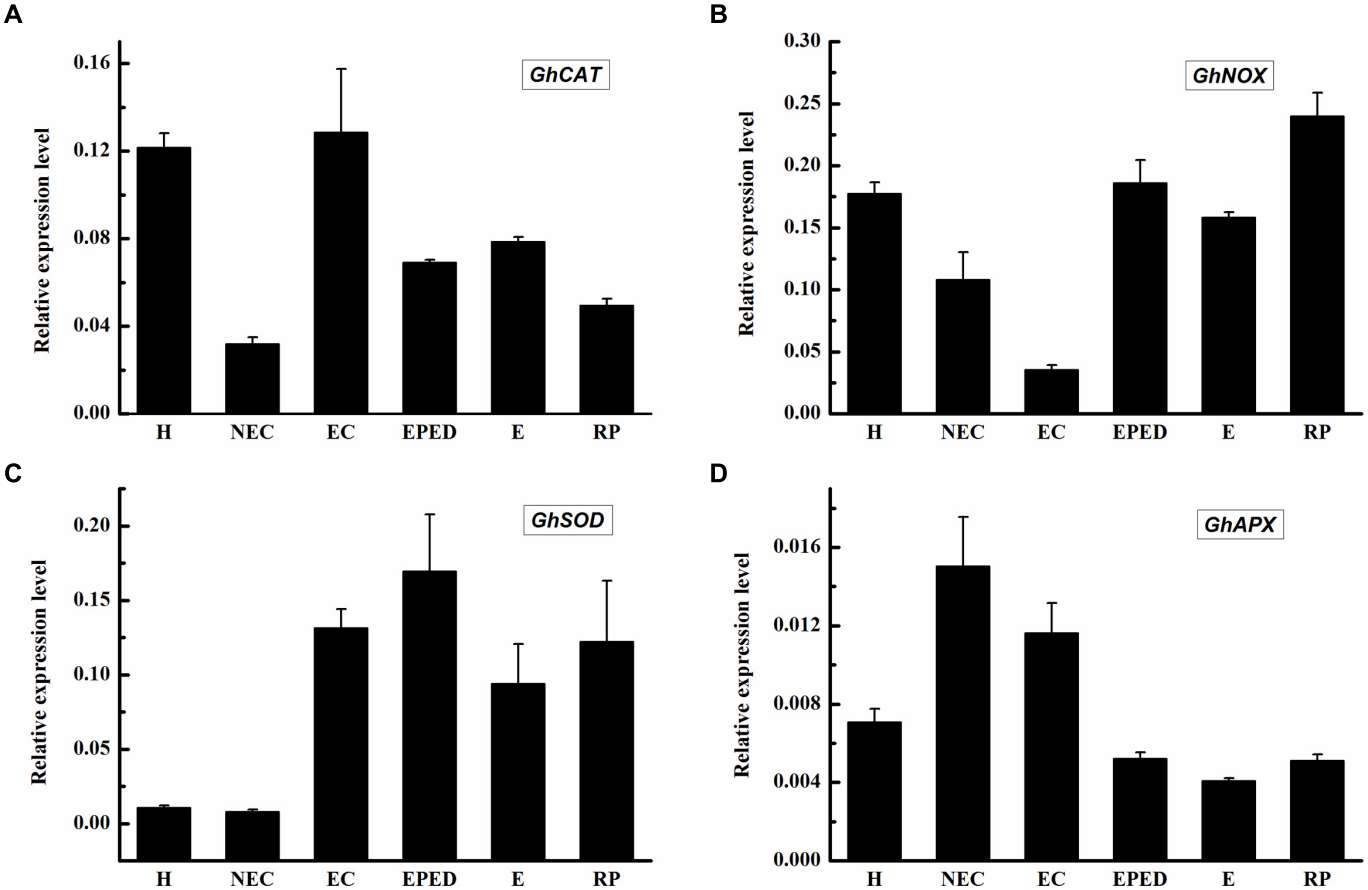
**Expression level of genes encoding *CAT, SOD, APX* and *NOX* during SE in ‘Xinluzao 33’.** The equation 2^-ΔCt^ was applied to calculate the relative expression level using *GhUBI* as the reference gene. **(A–D)** Relative expression level of GhCAT, GhNOX, GhSOD and GhAPX. **(A)** Relative expression level of GhCAT. **(B)** Relative expression level of GhNOX. **(C)** Relative expression level of GhSOD. **(D)** Relative expression level of GhAPX. The horizontal axis: H, Hypocotyl; NEC, Non-embryogenic callus; EC, Embryogenic callus; EPED, Early phase of embryo differentiation; E, Embryo; RP, Regenerated plantlets. Values are the mean + standard error (*n* = 3).

The expression levels of both *GhPAO1* and *GhPAO4* were quite low in the early phase of embryo differentiation even though H_2_O_2_ concentrations were high. This suggested that, in addition to the PAO-catalyzed PA pathway, other H_2_O_2_-generating pathways could also contribute to high H_2_O_2_ levels observed during cotton SE. These H_2_O_2_-generating pathways include the antioxidant enzyme system and the NADPH-dependent pathway. To address this issue, we analyzed the expression levels of genes encoding catalase (CAT), superoxide dismutase (SOD), NADPH oxidase enzyme (NOX), and ascorbate peroxidase (APX). The expression levels of *GhCAT*, *GhSOD* and *GhNOX* were all relatively highly expressed in the early phase of embryos differentiation, the somatic embryos, and the regenerated plantlets (**Figures 10A–C**). The highest expression level of *GhCAT* was observed in the embryogenic callus (**Figure 10A**). This was consistent with high H_2_O_2_ concentrations in these samples. The highest *GhAPX* expression was in the non-embryogenic callus (**Figure 10D**). Overall, these results support the hypothesis that all three H_2_O_2_-generating pathways contribute to high H_2_O_2_ concentrations during cotton SE.

## Discussion

Many studies have demonstrate the importance of PAs to SE in several plant species (Feirer et al., 1984; De-la-Peña et al., 2015). A wide range of possible roles of PAs in SE have been suggested. For example, it has been proposed that PAs (i) increase SE by regulating 2, 4-D synthesis and transport (Stuart and Strickland, 1984), (ii) enhance development of the cell cytoskeleton and (iii) generate energy required for cell division and differentiation during SE by interacting with Ca^2+^ transport (Kauss, 1987; Jansen et al., 1990). PAs have also been considered as hormone-like regulators that influence plant growth and development (Iqbal and Koenig, 1985). Experiments in which either PAs or auxin were exogenously applied demonstrated that PAs may have a synergistic role as auxin (Galston and Sawhney, 1990) to enhance the formation of somatic embryos (Trolinder and Goodin, 1988). It is important to note that PAs can be free, bound to macromolecules (DNA and protein), or attached (i.e., conjugated) to membranes (Kotzabasis et al., 1993). Different forms of PAs may have different functions during SE. Thermospermine is a structural isomer of Spm and it is assumed to be converted from spermidine by ACAULIS5 (ACL5). Genetic and molecular evidence indicates that thermospermine is produced through the action of *AtACL5* and required for stem elongation, xylem differentiation and vascular formation in *Arabidopsis thaliana* (Knott et al., 2007; Tong et al., 2014). In this study we analyzed for thermospermine but did not detect its presence during cotton SE.

Endogenous concentrations of Put, Spd, and Spm increased significantly from the embryogenic callus stage to the early stage of embryo differentiation (**Figures 1A–C**). Furthermore, H_2_O_2_ began increasing before the PAs increased (**Figure 4**). The increases in Put can be explained by dramatically increased expression levels of *GhADC1* and *GhADC2*, which are responsible for Put synthesis at the embryogenic callus stage and the early embryo differentiation stage (**Figure 2**). Although *ODC* seems to be active in cotton (Altman et al., 1982), the *GhODC* gene has yet to be identified; therefore its expression pattern was not analyzed in this study. Significant increases in PA biosynthesis in maize tumors were correlated with the transcriptional activation of the *ZmSAMDC2*, *ZmSAMDC3* and *ADC* genes (Rodríguez-Kessler et al., 2008). The changes in PA were related to an increase in the cell division rate. Exogenous application of Put, Spd, Spm, and H_2_O_2_ enhanced the growth and the development of somatic embryos, even when PA synthesis was blocked by D-Arg (**Figure 6**). These results not only confirmed observations reported in previous studies, but also revealed new findings about the roles of Put and H_2_O_2_ in the formation of cotton somatic embryos. Specifically, Put seemed to promote embryo formation whereas H_2_O_2_ seemed to promote cotyledonary embryos development.

In plants, H_2_O_2_ is considered to be one of the most important signaling molecules of abiotic stresses (Neill et al., 2002b). An extracellular oxidative burst is an early plant response to biotic/abiotic stress (Bolwell et al., 2002). Plant regeneration *via* SE is one of the best examples of plants under serious stress (Jayashree et al., 2003). A role of H_2_O_2_ in SE has been demonstrated in *Lycium barbaru* (Kairong et al., 1999) and *M. crystallinum* L. (Libik et al., 2005). However, most studies about the role of H_2_O_2_ in SE have focused on the antioxidant enzyme system. Relatively little attention has been paid to the role of H_2_O_2_ produced by the PA metabolism pathway. Endogenous H_2_O_2_ concentrations have been shown to be correlated with the activities of APX and CAT during SE of *Astragalus adsurgens* Pall (Luo et al., 2001). Similarly, the expression levels of *CAT*, *SOD* and *APX* were observed to be relatively high during SE in *L. leptolepis* (Zhang et al., 2010). In our study, *GhCAT*, *GhSOD* and *GhNOX* all seemed to have a role in H_2_O_2_ production during cotton SE (**Figures 10A–C**), whereas *GhAPX* did not (**Figure 10D**). These results suggested that the antioxidant enzyme system and the NADPH-dependent pathway both contribute to H_2_O_2_ synthesis during cotton SE. More importantly, we observed that active PAO was essential for healthy development of somatic embryos (**Figures 7** and **8**). *GhPAO1* and *GhPAO4* were highly expressed in the embryogenic callus (**Figure 9**), suggesting that H_2_O_2_ produced by the PAO metabolic pathway could be indispensable for the conversion of non-embryogenic callus to embryogenic callus during cotton SE.

Polyamine metabolism is one of the main sources of reactive oxygen species in plants under stress (Skopelitis et al., 2006). Many reactions in the PA metabolic pathway produce H_2_O_2_, especially the pathways metabolized by copper amine oxidase (CuAO) and flavin-containing PAO. Recent studies have cloned the gene(s) encoding PAO, purified the PAO enzyme, and investigated the spectral characteristics, subcellular localization and substrate specificity of PAO (Fincato et al., 2011; Ono et al., 2012; Liu et al., 2014). In plants, PAOs are preferentially associated with the primary and secondary cell walls of tissues undergoing lignification, suberization, and wall stiffening. Biochemical, histochemical, and immunocytochemical studies have confirmed the localization of PAO in primary and secondary cell walls of xylem parenchyma, the endodermis, and the epidermis of maize seedlings (Slocum, 1991). PA Oxidase5 (*AtPAO5*) regulates *Arabidopsis* growth and development through thermospermine oxidase activity (Kim et al., 2014). *OsPAO7* has been implicated in the biosynthesis of lignin, a major component of secondary wall thickening in anthers (Boerjan et al., 2003). It has been suggested that H_2_O_2_ generated by extracellular PAOs could underlie tissue differentiation due to the coordinated processes of cell wall maturation and programmed cell death (Desikan et al., 1998; Del Duca et al., 2014). Studies involving overexpression or down-regulation of apoplastic PAO indicate that H_2_O_2_ derived from PA catabolism is important in the induction of either salinity-induced tolerance or programmed cell death in tobacco (Moschou et al., 2008). In addition, a recent study showed that PAO activity could be modulated by interaction between PAs and a regulatory element, such as uORF, located in the 5′-untranslated region of the *AtPAO2* gene (Guerrero-González et al., 2014). Hence, it can be stated that a complex interplay of events modulates the levels of both AO and PAs. The rate of their secretion in the cell wall governs the spatio-temporal features of the AO-dependent biosynthesis of extracellular H_2_O_2_, which has been shown to play the dual role of (i) triggering peroxidase-mediated wall stiffening events and (ii) signaling the modulation of defense and hypersensitive response (HR)-cell death gene expression (Cona et al., 2006; Kärkönen and Kuchitsu, 2015; Mastracci et al., 2015). In this study, PAO activities significantly increased after the non-embryogenic callus stage (**Figure 7**). Repressing PAO activity by 1, 8-DO resulted in brown and necrotic cultures (**Figure 8B**) and a significant decrease in both FW and somatic embryo number (**Figures 8D,I**). Importantly, the negative effects of 1, 8-DO were reversed by application of exogenous H_2_O_2_ (**Figure 8C**). Consistent with our observation, cell wall-localized *Arabidopsis AtAO1* has been reported to be expressed in root cap cells and protoxylem precursors at early stages of vascular tissue differentiation (Sandip et al., 2015). In cell walls, CuAOs and PAOs share overlapping roles as H_2_O_2_ sources in developmentally- or light-regulated cell wall maturation events (Cona et al., 2005), as well as in oxidative bursts occurring during defense responses against biotic and abiotic stresses (Cona et al., 2006; Savatin et al., 2014) such as pathogen attack, salt stress and wound healing (Tisi et al., 2008; Mitsuya et al., 2009; Rodríguez et al., 2009; Mastracci et al., 2015). Our results indicate that active PAO and H_2_O_2_ are both essential in cotton SE, which is a severe biotic stress. To our knowledge this is the first report about the role of PAO in the conversion of EC into somatic embryos.

Nitric oxide is another signal molecule involved in the PA metabolic pathway; however, it is not known if NO has a role in SE. One previous study indicated that endogenous NO emission decreased significantly during the early stages of SE of *Araucaria angustifolia* treated with glutathione (Vieira et al., 2012). In contrast, Put increased NO emission in embryogenic suspension cultures of *A. angustifolia (*Silveira et al., 2006*)*. The addition of SNP, a donor of NO, increased cell elongation and the expression of the receptor kinase (SERK) protein during SE of *Medicago sativa* L (Ötvös et al., 2005). In our study, NO concentrations tended to decrease during cotton SE; however, the declines were not very large (**Figure 4A**). Furthermore, cotton SE was completely inhibited when 1 mM SNP was added to the somatic embryo induction medium (**Figure 4C**). The function of NO-related chemicals and genes involved in NO-PA metabolism should be studied in the future.

**FIGURE 11:**
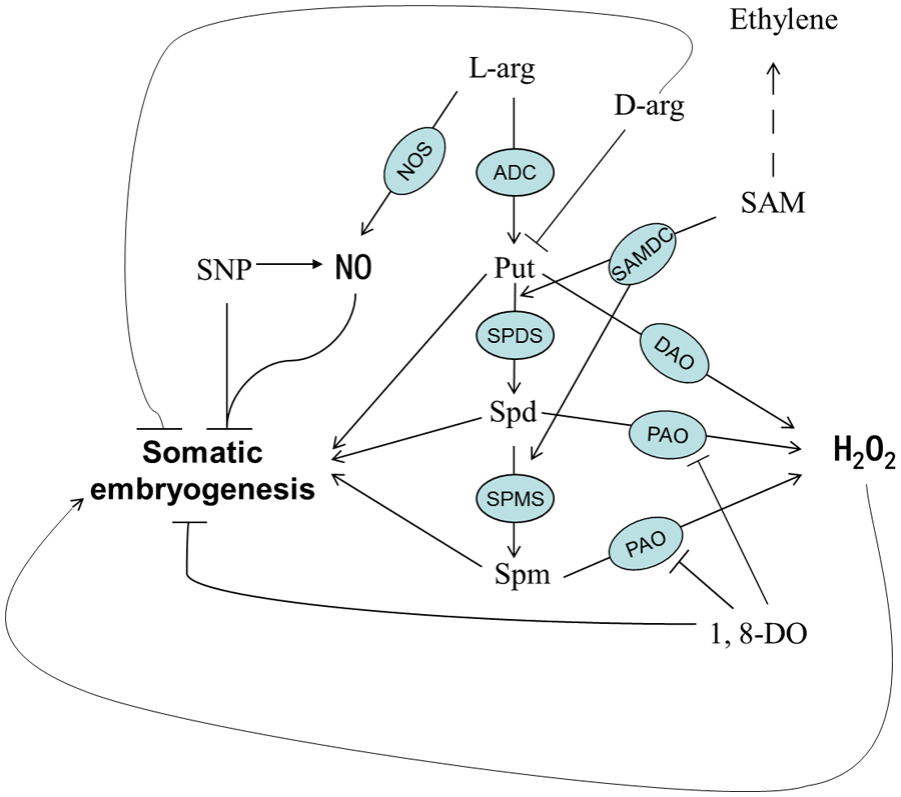
**A proposed model of PA metabolism and possible involvement of PA-related chemicals during SE in cotton**.

Based on previous knowledge and the results of this study, we developed a working model showing the relationship between SE and chemicals involved in PA biosynthesis (**Figure 11**). PAs and H_2_O_2_ are the key components of this model and are essential for normal SE. The differentiation and the development of embryonic callus are both inhibited when the synthesis of PAs is suppressed or PAO activity is blocked. The H_2_O_2_ detected during SE could have been generated from multiple pathways; however, the PA metabolic pathway and PAO seem to be critical factors affecting cotton SE.

## Author Contributions

Conceived and designed the experiments: W-HC, H-GZ, and JS Performed the experiments: W-HC, F-LW, and X-QC. Analyzed the data: W-HC. Contributed reagents/materials/analysis tools: JS, Y-QS. Wrote the paper: W-HC, Q-HZ.

## Conflict of Interest Statement

The authors declare that the research was conducted in the absence of any commercial or financial relationships that could be construed as a potential conflict of interest.
