# Supplementary material for: Polyamine and Its Metabolite H2O2 Play a Key Role in the Conversion of Embryogenic Callus into Somatic Embryos in Upland Cotton (Gossypium hirsutum L.)
Source: Front Plant Sci. 2015 Dec 2;6:1063. doi: 10.3389/fpls.2015.01063 (PMC4667013; doi:10.3389/fpls.2015.01063)
Supplement: Supplementary file 1 [file Data_Sheet_1.PDF]

Supplementary Material

Polyamine and its metabolite H_2O_2 play a key role in the conversion of embryogenic callus into somatic embryos in upland cotton (*Gossypium hirsutum* L.)

Wen-Han Cheng1, Fan-Long Wang1, Xin-Qi Cheng1, Qian-Hao Zhu2, Yu-Qiang Sun1, 3, Hua-Guo Zhu1*, Jie Sun1*

* Correspondence: Jie Sun, sunjie@shzu.edu.cn; Hua-Guo Zhu, zhgroger@sohu.com.

Three supplementary figures and three supplementary tables included in this document.

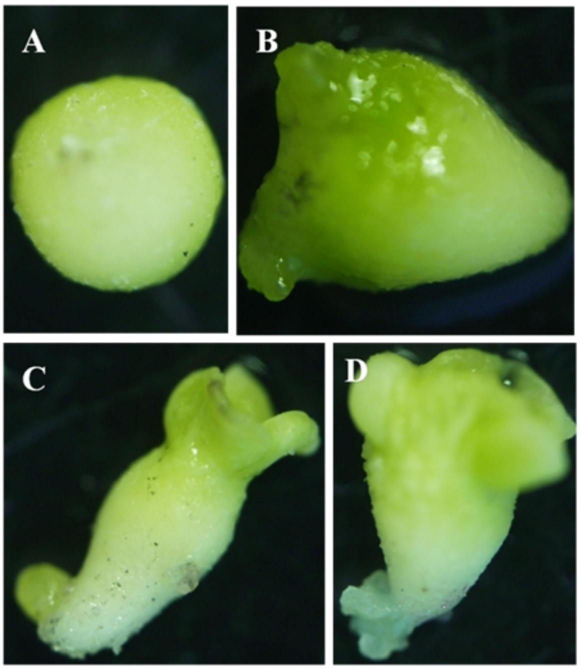

Figure S1 Stages of somatic embryo in Xinluzao 33 (**A**) Globular embryo. (**B**) Heart-shaped embryo. (**C**) Torpedo-shaped embryo. (**D**) Cotyledonary embryo.

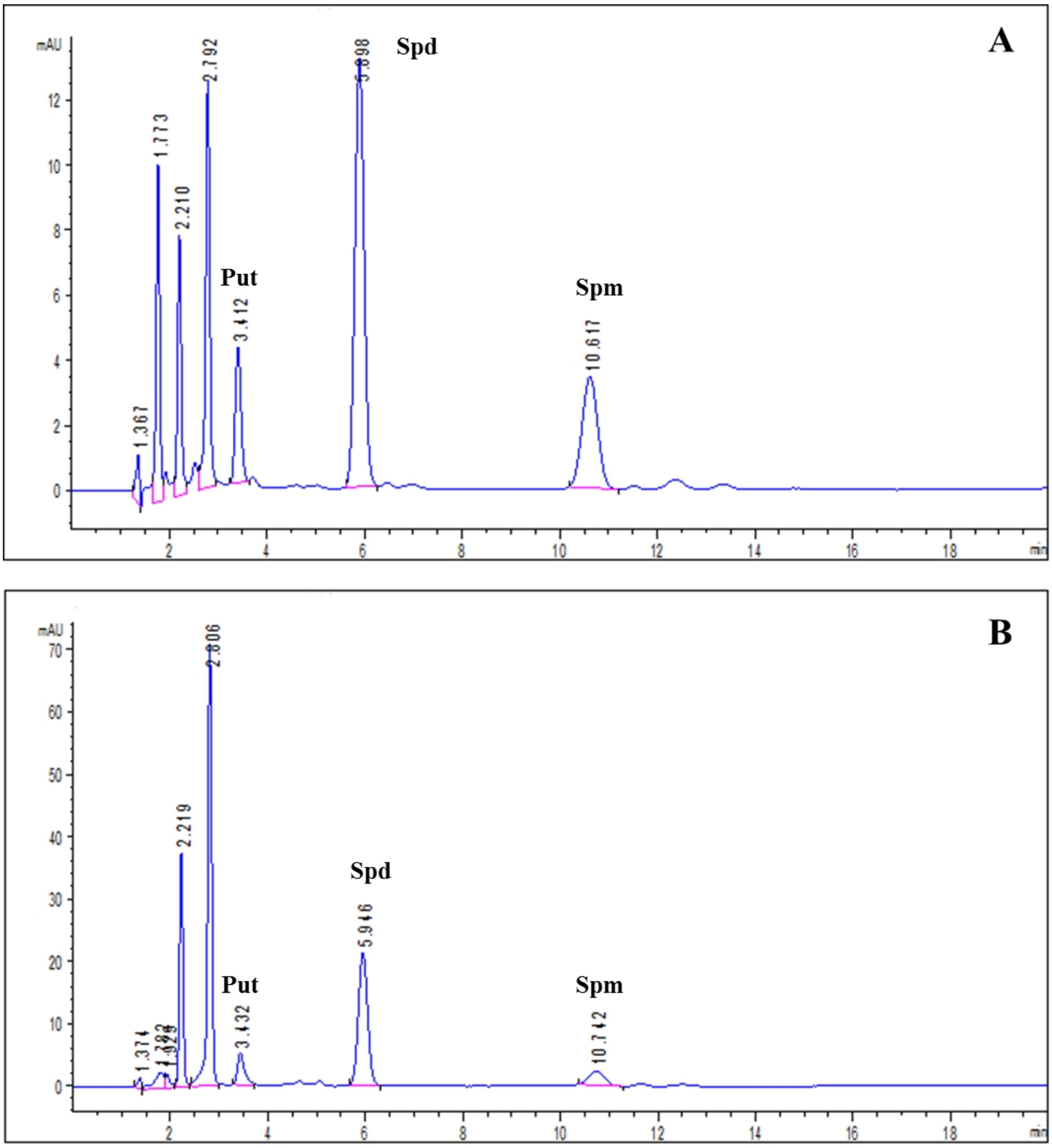

Figure S2 Comparison of peaks of the standard PAs and the sample measured using the Aglient 1200 system. (**A**) Standard PAs. (**B**) Sample.

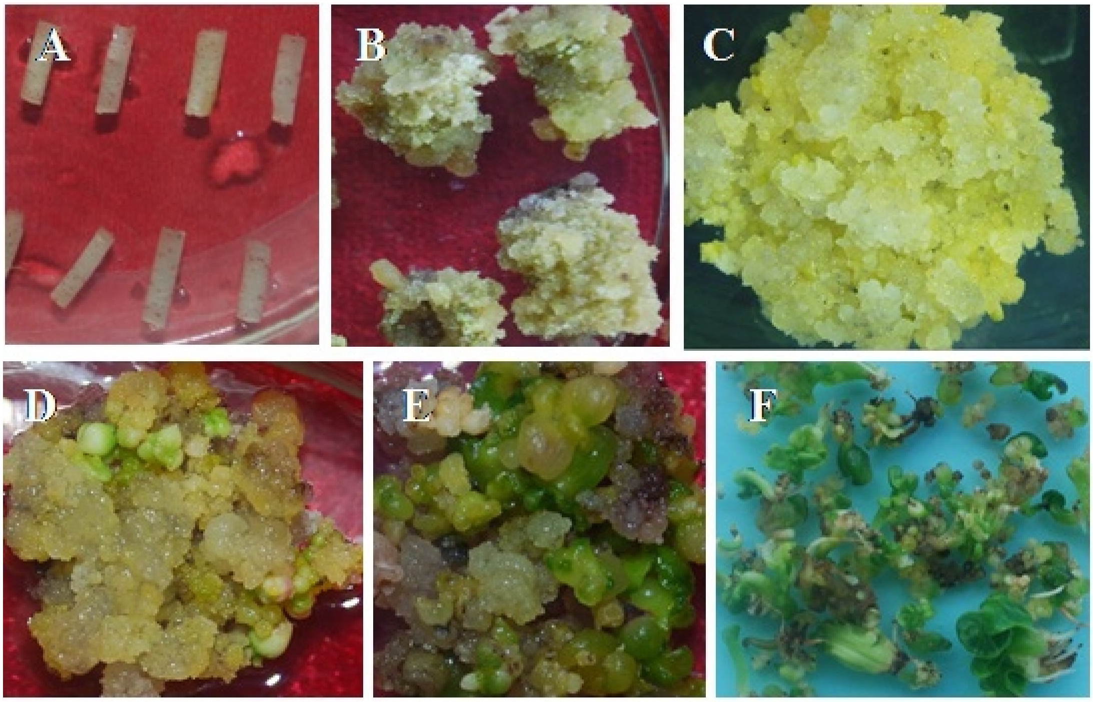

Figure S3 Different development stages of somatic embryogenesis in Xinluzao33. (**A**) Hypocotyl. (**B**) Non-embryogenic callus. (**C**) Embryogenic callus. (**D**) Early phase of embryo differentiation. (**E**) Embryos. (**F**) Regenerated plantlets.

Table S1 Media used in cotton SE

No.	Media name	Abbreviations	MS/MSB	Glucose (g /L)	Agar (g /L)	Phytagel (g /L)	2,4-D (mg /L)	KT (mg /L)	IBA (mg /L)	Other
**1**	**Aseptic media for seedling**	**AMS**	1/2MS	15	8	-	-	-	-	-
**2**	**Callus induction media**	**CIM**	MSB	30	-	2.5	0.1	0.1	-	-
**3**	**Embryogenic callus induction media**	**ECIM**	MSB	30	-	2.5	0.05	0.1	-	-
**4**	**Embryogenic callus proliferation media**	**ECPM**	MSB	30	-	2.5	-	0.15	0.5	MSB with no NH_4NO_3, but double KNO_3.
**5**	**Somatic embryo induction media**	**SEIM**	MSB	30	-	4	-	0.15	0.5	-
**6**	**Media for regenerated plants**	**MRP**	1/2MSB	15	-	2.5	-	-	-	-
**7**	**Liquid somatic embryo induction media**	**LSEIM**	MSB	30	-	-	-	0.15	0.5	1g/L NaCl

Table S2 Primers used in qRT-PCR

Gene	Gene ID	Forward primer	Reverse primer	Amplicon length (bp)
GhPAO1	XM_012618258.1	GAGTTACATTTAACAGCGAAGA	CTGGATAATCGCCTCAAA	219
GhPAO2	XM_012581927.1	CCAGTTATGGGTGAGGAA	CGCTACTTGCATTGGTTT	171
GhPAO3	XM_012588914.1	GAGTGTCGGTGCGTTTCT	TTACGAGGCAGAAAGCGA	195
GhPAO4	XM_012620491.1	ATGGCTGAACCATTACCTAC	TTAGTGGTATTAGGGAAGCC	129
GhADC1	XM_012611440.1	GAAGTCTTGCTCCGGTTGT	CTACGGCAGCAGCATCTAC	205
GhADC2	XM_012624962.1	CGCTGCTTCCTTTTGAGG	GCTTGACTCGGAATTAGCC	259
GhADC3	XM_012605083.1	CGAAGTGTTGGGTAGTATCGT	ACCGTTGTGGCAATGGAG	271
GhSAMDC1	XM_012623384.1	GAAAACAAAAACATAAAACCATAA	GTAAAAGGATACCGTTGCG	214
GhSAMDC2	XM_012607973.1	GGTATTTCCTTGGGCGACTT	TGGGCTCTTGACAATGATCTAA	143
GhSAMDC3	XM_012617948.1	AAGGTGAGGGACCAGAAGGAT	AGACTAACTGAAGAGCCAAA	230
GhSAMDC4	XM_012631940.1	TAACACCATCAAATCCCACC	AAGTTCCAACCGCTTCTCAA	168
GhSPDS	CL1916.Contig3_All	TGAGGCTAAATGCCATTCTA	ACAAGAACCTCTTGATAGT	177
GhSPMS	CL1916.Contig5_All	ATCAGACTATCAAGAGGTC	CCAACAACCAGAACAGT	168
GhSOD	CL8467.Contig1_All	GGAAGAGCTGTAGTTGT	GCCTTGCAGACCAATA	120
GhCAT	CL2833.Contig1_All	ATGTGAAATTCCATTGGAAACCCA	TCAAACCTATCCTCATGAAG	187
GhAPX	CL5637.Contig1_All	AGAACTGTGCTCCACTCATGCT	GTCAGCGTATGAAAGGATA	197
GhNOX	CL554.Contig2_All	GCCTCCTTAGAGCTGATT	AATGCTGATCATGGGGGTT	158
GhNOS	Unigene26726_All	AGTCGGCTCAGAGGTCAA	TACCCATTCATGCTGTGC	187
GhUBI	XM_012634824	CAGATCTTCGTCAAAACCCT	GACTCCTTCTGGATGTTGTA	209

Table S3 Nucleotide sequences of *SPDS, SPMS, CAT, NOX, SOD, APX* and *NOS* based on the transcriptomics of upland cotton Xinluzao 33.

Gene ID	Gene name	Nucleotide sequences
>CL5637.Contig1_All	*GhAPX*	ATGACCAAGTGTTACCCAACCGTTAGCGAGGAGTACCAAAACGCCGTTCAAAAGGCTAAGAGGAAGCTAAGAG<br>GTCTCATCGCTGAGAAGAACTGTGCTCCACTCATGCTCCGTCTAGCGTGGCACTCAGCTGGAACTTTTGATGTCA<br>AGACCAAGACCGGAGGTCCATTCGGAACCATGAAGCAACCTGCTGAGCTCGCTCATGCTGCTAACAACGGTCTC<br>GATATTGCAGTCAGGCTTCTCGAGCCGATCAAGGAGCAGTTCCCTATCCTTTCATACGCTGACTTCTATCAGCTT<br>GCTGGTGTTGTTGCTGTTGAGATCACCGGTGGGCCTGAAGTTCCATTCCATCCTGGAAGAGAGGACAAGCCTCA<br>CCCACCACCTGAGGGTCGTCTTCCCAATGCTACTGAGGGAGCTGATCACTTGAGGCAGGTGTTTAGTCATCAAA<br>TGGGTCTTAGCGACCAGGACATTGTTGCTCTTTCTGGTGGCCACACCCTGGGAAGGTGCCACAAGGAGAGGTCC<br>GGGTTTGAGGGACCATGGACTACCAACCCTCTTATCTTTGACAACTCTTACTTCAAGGAGCTTTTGTCGGGAGAG<br>AAGGAAGGCCTTCTTCAGCTACCAACTGACAAAGTTCTCCTGTCAGATCCTGTTTTCCGTCCATTGGTTGAAAAA<br>TATGCTGCCGATGAGGATGCCTTCTTTGCTGACTACACCGAAGCTCACCTGAAGCTCTCTGAGCTAGGATTTGCT<br>GATGCA
>CL8467.Contig1_All	*GhSOD*	GGAAGAGCTGTAGTTGTCCATGCAGATCCCGATGACCTTGGCAAGGGCGGCCATGAGCTCAGCAAAAGCACAG<br>GAAATGCTGGCGGCAGAGTAGCTTGCGGTATTATTGGTCTGCAAGGC
>CL554.Contig2_All	*GhNOX*	CCATTTGAATGGCATCCATTTTCGATTACTTCTGCCCCTGGAGATGACAATCTCAGTGTTCATATAAGGACTCTC<br>GGTGATTGGACACGACAACTTAGGACCGTATTTTCAGAGGTGTGTCAACAACCTACTGATGGGAAAAGTGGCCT<br>CCTTAGAGCTGATTGCTTGCAAGGAACTAACAACCCAAATTTCCCACGAGTTCTAATCGACGGACCATACGGAG<br>CACCGGCACAAGACTACAAGAAATACGAAATCGTTTTGCTAATCGGATTGGGGATCGGAGCAACCCCCATGAT<br>CAGCATT
>CL2833.Contig1_All	*GhCAT*	CCTGATATGGTCCATGCTCTTAAACCAAACCCGAAGTCCCACATTCAGGAAAACTGGAGGATTCTGGACTTCTT<br>TTCGCACCATCCCGAAAGCTTGCACATGTTCACCTTCCTGTTTGATGACATCGGTGTTCCACAGGATTATAGGCA

		CATGGATGGCTCTGGTGTTCATACCTACACACTAATCAACAAGGCTGGGAAGTCACACTATGTGAAATTCCATT<br>GGAAACCCACTTGTGGTGTAAAATCTTTGTTGGAGGATGAAGCCATAAGAGTAGGTGGTGCGAATCACAGTCAT<br>GCCACACAAGATCTTTATGACTCGATTGCAGCCGGAAACTACCCTGAATGGAAACTGTTCATTCAAATAATGGA<br>TCCTCTTCATGAGGATAGGTTTGATTTTGACCCACTTGATGTAACCAAGACATGGCCAGAGGACATCTTCCCATT<br>GCAGCCGGTGGGTCGAATGGTGTTAAACAAGAACATCGATAACTTCTTTGCTGAGAACGAGCAATTAGCCTTCT<br>GCCCTTCCCTCATTGTCCCTGGCATCTATTATTCAGATGATAAGTTGCTTCAAACTAGAATCTTCTCCTATTCTGA<br>CACTCAAAGGCACCGTCTTGGACCAAATTATCTGCAGCTTCCAGCTAATGCTCCCAAATGTGCTCATCACAATA<br>ATCACCATGAAGGTTTTATGAATTTTATGCATAGAGATGAGGAGGTGAACTACTTCCCTTCGAGGTACGATCCT<br>GTTCGCCACGCTGAGAAGCACCCTATTCCTAGCACCGTTTACAGTGGCAAAAGAGAGAAGTGCATTATCGGTAA<br>GGAGAACAACTTTAAGCAGCCTGGGGAACGATACCGGTCCTTTTCAGCTGACAGGCAAGAACGGTTTATTAATC<br>GCTGGATTGATGCATTGTCTGATCCAAGGGTCACACACGAGATCCGCAGCATTTGGATCTCATACTGGTCTCAG<br>GCT
>CL1916.Contig3_All	*GhSPDS*	ATGGGGGAAGACGCAGGAAGAGGTTTGGAATGCCAGAAGATTATGGATGGGAAGGGGAATAATGGGAATAGT<br>TCACAGAAGGCTATCCCTTCTTGTTGCTTGAAGGCTAGGGCTTCAGCCCCTGAGCTTGAGGCTAAATGCCATTCT<br>ACCGTTGTTTCTGGGTGGTTCTCAGAATCTCAGTCTTCTTCTGATAAGGCCGGTAAAATGGTTTACTTCAACAAT<br>CCTATGTGGCCCGGAGAAGCACATTCTCTGAAAGTTGAAAGCATTTTGTACAAAGCAAGGTCAGACTATCAAGA<br>GGTTCTTGTTTTTGAGTCATCAAGCTATGGGAAAGTGCTTGTTCTTGATGGTATAGTTCAGCTGACTGAGAAGGA<br>TGAATGTGCATATCAGGAGATGATAACTCATCTTCCCCTTTGCTCAATTCCATCTCCCAAAACTGTAATC
>CL1916.Contig5_All	*GhSPMS*	ATGGGGGAAGACGCAGGAAGAGGTTTAGAATGCCCGAAGCTTATGGATGGGAAGGTGAATAGTGGTAATGGCT<br>CAGAGAAGGTTATCCCTTCTTGTTGTTTGAAGGCCAGGGCTTCAGCCCCTGAGCTTGAGGCAAAATGCCATTCT<br>ACTGTTGTTTCTGGGTGGTTCTCGGAATCTCAGTCTTCCACTGATGATGCTGGTAAAATGGTCTACTTCAACAAT<br>CCTATGTGGCCGGGAGAAGCGCATTCTCTGAAAGTTGAAAGCATTTTATTCAAGGAAAAATCAGACTATCAAGA<br>GGTCCTTGTTTTTGAGTCATCGACATATGGGAAAGTGCTTGTTCTTGATGGTATAGTTCAGCTGACTGAGAAGGA

		TGAATGTGCATATCAGGAGATGATTGCTCATCTTCCACTTTGTTCAATTCCATCTCCGAAAACTGTTCTGGTTGTT<br>GGAGGTGGTGATGGTGGAGTTCTTAGGGAGATTTCTAGACACAGCTCTGTGGAGCACATTGATATATGTGAAAT<br>AGATAAGATGGTTATAGATGTTTCCAAGAAGTTCTTTCCAGAATTAGCTGTTGGATTTGAGGACCCTCGCGTCA<br>ATCTTCATGTTGGTGATGCTATTGAGTTTCTTCGTCATGCTCCAAAAGGAAAGTATGATGCAATTATTGTTGATT<br>CATCAGATCCTGTAGGTCCTGCTCAAGAGCTTGTAGAGAAACCATTTTTTGAGACTATAGCTCAAGCATTAAGG<br>CTTGGTGGTGTCCTTTGTAACATGGCAGAGAGTATGTGGCTTCATACACATTTAATTGAAGATATGATTTCTATT<br>TGCCGTGAGACATTCAAGGGGCCTGTTCATTATGCATGGGCAAGTGTTCCAACATATCCAAGTGGTGTGATTGG<br>TTTTTTGTTATGTTCCACGGAGGGGCCACCAGTTGATTTTTTGAATCCTATAAACCCTATTGAGAAGCTAGACGG<br>TGCTTACCTTCATAAGAGAGAACTTAGATTTTACAACTCTGAGATGCATAGGGCTGCTTTTGCATTGCCTTCGTT<br>CCTGAAGAGGGAGGTGAGGCTACTATGAGACTCCACAGCCCCGGAACAAGAAAAC
>Unigene26726_All	*GhNOS*	ATGGCGCTCAAAACCCTTTCCACATTTCTCTCTCCTCTCCCTCTTCCGAAGCACTACACTCTCTCTATCTTCAACC<br>CCGTATCCCTTAATATTTACAGAAAATCCACTCCCCTTTCCTGCAAATCCACTCACTCGCACTCCCCATCTCAGTT<br>GCCACTCTCCGATACGAAATTGCTTTCTTCCGAACCGGAAGGAACTGGTGCGGCTTCTCCGACCCGGGGAGACC<br>GGTTTCTCGAAAGGCAACAGGCTGACGAGGCTGCCAAGCTTCTTATAAAGGAGATAAAGAAGACTAAGAAGAA<br>GACGAAGAAGGTTTTGAAGGTTAATACGGTAACCGCCTGTTGTTATGGTTGCGGAGCTCCATTGCAGACTTCGG<br>AGGTGGATGCTCCGGGTTATGTGGATATGGACACCTATGAATTGAAGAAGAAACACCACCAGCTAAGAACGAT<br>TCTTTGTGGGAGATGTAGGCTTTTATCTCATGGGCATATGATCACTGCTGTTGGTGGGAATGGAGGTTATTCTGG<br>GGGGAAGCAGTTTGTTTCAGCTGATGAGCTTCGGGAAAAGCTCTCTCACTTGCGACATGAGAAGGCTTTGATTG<br>TTAAATTGGTTGATATTGTGGACTTCAATGGCAGCTTTTTATCTCGTGTGCGTGATCTTGCTGGTGCAAATCCTAT<br>AATACTAGTTGTGACTAAGGTTGATCTTCTTCCAAAAGGGACTGATTTTAATTGTGTGGGTGATTGGGTTGTGGA<br>GGCCATTACAAAGAAGAAGCTTAATGTCTTGAGTGTGCATCTTACTAGCTCAAAGTCTTTAGTAGGGATTGCTG<br>GAGTTGCATCAGAAATCCAAAAGGAGAAGAAGGGAAGGGATGTGTACATTCTGGGATCAGCAAATGTGGGAA<br>AATCTGCATTCATAAGTGCCTTACTAAAAATGATGGCACAAAGGGATCCAGCAGCTGCAGCGGCACAAAAGTA

		CAAACCAATACAATCAGCTGTTCCCGGAACTACTTTAGGTCCAATCCAGATTGATGCTTTTCTTGGAGGAGGGA<br>AATTATATGATACACCAGGGGTTCATCTTCACCATAGGCAAGCTGCAGTAGTCCACTCAGAAGATTTACCTATC<br>CTTGCTCCTCAAAGTCGGCTCAGAGGTCAATCATTCCCTGTCTCCTCCAAAAATGGGATGGCAGGGAAATTCAA<br>TTCCAATGGCTTAAATGGGTTCTCAATATTTTGGGGTGGCCTTGTCAGAATTGATGTCTTGAAGGTTCTCCCTGA<br>GACATGTTTGACATTTTATGGGCCAAAGAAGTTACCCATTCATGCTGTGCCTACTCATGAAGCAGATGAATTTTA<br>CAAGAAAGAACTTGGAGTACTGTTGACACCACCAACTGGAAAAGATCGAGCAGGTGAATGGAGAGGACTGGA<br>GACAGTGCAGCAACTGCAAATAAACTTTGAGGATGCTGAAAGACCTGCTAGTGATGTGGCTATTTCAGGACTGG<br>GGTGGATCACCGTTGAACCGAGACGCGAGTCACTTGGAATATCTGAAAGTAATTTTTCAGAAACAACCAAACA<br>ATTGGATATTGTGGTCCATGTACCCAAGCCAGTTGAGATTTTCGTTCGGCCTTCAATACCCGTCGGTAAAGCTGG<br>AGCAGAATGGTACCAATATCGTGAGTTAACAGAAAAGGAAGAAGAAATA<br>AGACCAAAGTGGTACTTT
